# Skipping without and with hurdles in bipedal macaque: global mechanics

**DOI:** 10.1242/jeb.246675

**Published:** 2024-03-28

**Authors:** Reinhard Blickhan, Emanuel Andrada, Eishi Hirasaki, Naomichi Ogihara

**Affiliations:** ^1^ Science of Motion, Friedrich-Schiller-University, 07749 Jena, Germany; ^2^ Institute of Zoology and Evolutionary Research, 07743 Jena, Germany; ^3^ Center for the Evolutionary Origins of Human Behavior, Kyoto University, Inuyama, Aichi 4848506, Japan; ^4^ Department of Mechanical Engineering, Keio University, 3-14-1 Hiyoshi Kohoku-ku, Yokohama 2238522, Japan; ^5^ Department of Biological Science, Graduate School of Science, The University of Tokyo, 7-3-1 Hongo Bunkyo-ku, Tokyo 113-0033, Japan

**Keywords:** Macaque locomotion, Gait, Leg mechanics

## Abstract

Macaques trained to perform bipedally used running gaits across a wide range of speeds. At higher speeds they preferred unilateral skipping (galloping). The same asymmetric stepping pattern was used while hurdling across two low obstacles placed at the distance of a stride within our experimental track. In bipedal macaques during skipping, we expected a differential use of the trailing and leading legs. The present study investigated global properties of the effective and virtual leg, the location of the virtual pivot point (VPP), and the energetics of the center of mass (CoM), with the aim of clarifying the differential leg operation during skipping in bipedal macaques. When skipping, macaques displayed minor double support and aerial phases during one stride. Asymmetric leg use was indicated by differences in leg kinematics. Axial damping and tangential leg work did not influence the indifferent peak ground reaction forces and impulses, but resulted in a lift of the CoM during contact of the leading leg. The aerial phase was largely due to the use of the double support. Hurdling amplified the differential leg operation. Here, higher ground reaction forces combined with increased double support provided the vertical impulse to overcome the hurdles. Following CoM dynamics during a stride, skipping and hurdling represented bouncing gaits. The elevation of the VPP of bipedal macaques resembled that of human walking and running in the trailing and leading phases, respectively. Because of anatomical restrictions, macaque unilateral skipping differs from that of humans, and may represent an intermediate gait between grounded and aerial running.

## Teaser

**Summary:** During fast bipedal locomotion macaques prefer to skip; the leg torque differs between the trailing and leading leg but not the transmitted impulses. Double support is crucial for the jump.

## INTRODUCTION

In the wild, macaques prefer to locomote quadrupedally (Chatani, 2003; Fiers et al., 2013). The individuals trained at the Suo Monkey Performance Association, Japan, learned to pose and locomote bipedally while guided on a leash. They walked along the theater, but they never seemed to run with aerial phases. By investigating their running ability, we discovered that macaques were able to use a variety of gaits such as walking, grounded running (running gait without aerial phases), aerial running (with two aerial phases) and hopping (Ogihara et al., 2010, 2018). We also found that macaques preferred to bounce instead of vaulting over stiff legs at Froude speeds above 0.4 and used grounded running across a wide range of speeds (Ogihara et al., 2018; Blickhan et al., 2018, 2021), even though humans usually avoid this gait because it is seemingly energetically more expensive than aerial running (Bonnaerens et al., 2018; Rummel et al., 2009). The compliant legs of macaques facilitated this gait (Andrada et al., 2020). Despite some morphological adaptations to bipedal walking, such as a human-like lordosis and more robust femora (Nakatsukasa et al., 2006), restricted hip joint extension (Ogihara et al., 2007) in particular enforces a crouched leg posture (Blickhan et al., 2021) and high leg compliance (Blickhan et al., 2018).

**Fig. 1.**
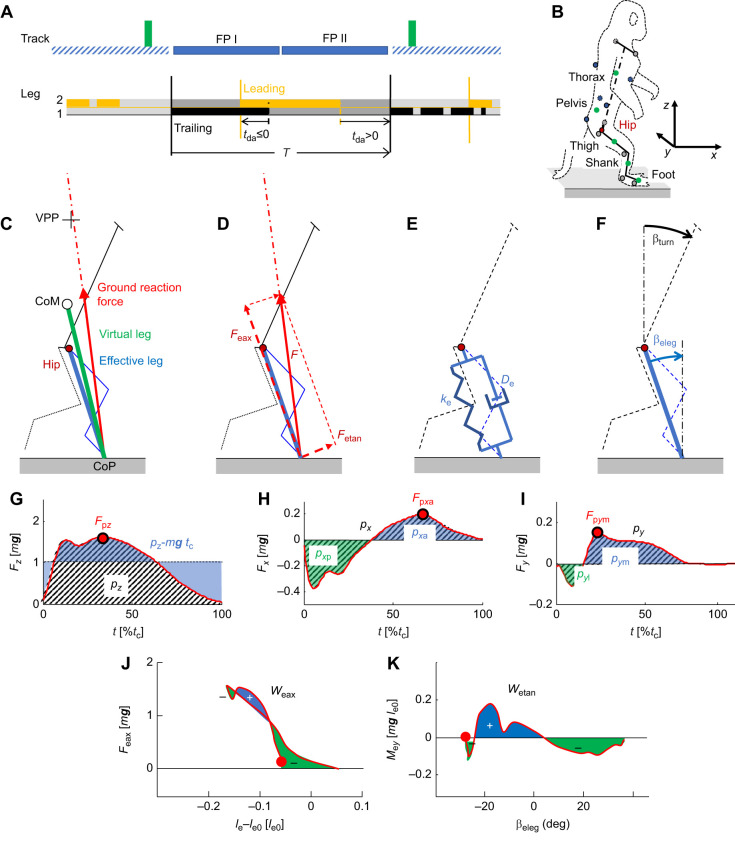
**Setup and definitions.** (A) Setup. The macaques crossed a track with two force plates (FP I, FP II). During hurdling, two hurdles (green rectangles) were placed before and after the force plates. Below: the stepping pattern during skipping and hurdling entails both a double support period (*t*_da_≤0) and an aerial (*t*_da_>0) phase, defining the leading and trailing leg. The gait cycle usually starts with the touch down (solid vertical lines) of leg one, the trailing leg, onto the first force platform followed by the touch down of leg 2, the leading leg, on the second force plate with a double support until lift off (dashed vertical lines) of the trailing leg. The aerial period starts with the lift off of the leading leg. The stride ends with the touch down of the trailing leg after the flight phase. During four skipping trials, the macaques stepped onto the first platform with the leading leg. (B) System of co-ordinates *x*,*y*,*z*. The center of mass (CoM) of the segments (green circles), hip position (red circle) and the CoM of the macaque were calculated based on attached reflective markers (blue and grey circles). (C) The effective leg connects the center of pressure (CoP) and the hip, the virtual leg the CoP and the CoM. The direction of the ground reaction force vectors crossed at the virtual pivot point (VPP). (D) The ground reaction force (*F*) was decomposed in an axial (*F*_eax_) and tangential (*F*_etan_) force component. Depicted for the effective leg. (E) The force–length characteristics of the axial leg were approximated by a spring (*k*_e_)–damper (*D*_e_) element. (F) Tilt of the trunk (β_trunk_) and leg angle (β_leg_). (G) Vertical component of the ground rection force [*F_z_*(*t*)], with its peak value (*F*_p*z*_; red circle), vertical impulse (*p_z_*; hatched) and *p_z_*−*m**g**t*_c_ (blue). *t*_c_, contact time. (H) Posteri-anterad component of the ground reaction force [*F_x_*(*t*)], with its peak anteriad value (*F*_p*x*a_; red circle), the total impulse (*p_x_*; hatched), and the posteriad (*p_x_*_p_; green), and anteriad (*p_x_*_a_; blue) contributions. (I) Lateri-mediad component of the ground reaction force [*F_y_*(*t*)], with its peak mediad value (*F*_p*x*m_; red circle), the total impulse (*p_y_*; hatched) and the laterad (*p_y_*_l_; green) and mediad (*p_y_*_m_; blue) contributions. (J) Axial force of the effective leg [*F*_eax_(*l*_e_−*l*_e0_)] in dependence on the change in leg length and the axial work (*W*_eax_), with its positive (blue) and negative (green) contributions. (K) Hip moment of the effective leg [*M*_e*y*_(β_eleg_)] in dependence on the leg angle and the tangential work (*W*_etan_), with its positive (blue) and negative (green) contributions.

As in children of the age of about 5 years (Roncesvalles et al., 2001), the bipedal macaques used unilateral skipping (bipedal galloping) when guided for fast locomotion (Ogihara et al., 2018). Unlike in a trotting quadruped (e.g. horse) and a running biped (e.g. human), where the left and right legs operate out of phase (phase shift 50%, symmetrical gait), during quadrupedal gallop (horse) and bipedal skipping (human), contralateral legs move more in phase (asymmetrical gait; Hildebrand, 1989). In unilateral skipping (bipedal galloping), the left and right legs alternate from step to step as during running but with a shifted phase between the contralateral legs. Skipping in general is characterized by a sequence of double support and flight phase. After the aerial phase the subject lands with the trailing leg. Towards the end of the contact of the trailing leg, the leading leg touches down second, resulting in a double support phase. With this leading leg the subject takes off to the aerial phase (Fig. 1A,B). In unilateral skipping, the same leg for all strides constitute either the leading or the trailing leg. In contrast, during bilateral skipping (‘high knee skips’), the two legs switch their roles from stride to stride. Adult humans avoid skipping and prefer the metabolically cheaper walking or running (Fiers et al., 2013). Nevertheless, like running, skipping can be self-stable and quite robust against disturbances (Andrada et al., 2016; Müller and Andrada, 2018). In macaques, during their preferred quadrupedal locomotion, the trot–gallop transition speed is shifted for the hindlimbs with respect to the forelimbs (Vilensky, 1983), indicating a weak neuronal coupling. The transverse quadrupedal gallop preferred in the wild (Kimura, 1992; Nakatsukasa et al., 2006, 2004) may preadapt the macaque for the frequently used bipedal galloping. Bipedal galloping seems to represent a quite natural gait. In human skipping, the coordination at the hip joint enforces a differential operation of trailing and leading legs, facilitating skipping (Pequera et al., 2021). We assume that, despite of the limited hip extension, the compliant legs identified in macaques (Blickhan et al., 2018) allow for a differential function of the leading and trailing leg.

In their performances, the macaques demonstrated their jumping ability traversing single hurdles of up to 2 m height. However, during skipping, they hardly left the ground. In order to test whether musculoskeletal limitations combined with the demands of coordination during skipping prevent a more dynamical gait, we placed low hurdles on the track at the distance of a stride. The macaques chose to skip seemingly effortlessly across these hurdles, taking the double support in between. Skipping across hurdles was expected to accentuate the dynamics and potential differences in the operation of the trailing and leading leg.

In order to understand the differential dynamical function of the leading and trailing legs, the ground reaction forces in skipping can be investigated by comparing peak values and form parameters used to describe distributions in statistics (skew and kurtosis; e.g. Blanca et al., 2013; Hedderich and Sachs, 2016) as well as the generated impulses which are relevant for describing the changes in velocity of the center of mass (CoM). The task of the trailing leg could be to accommodate the fall from the preceding flight phase and that of the leading leg to accelerate to generate the next flight phase. We assumed that the two legs generate a similar change in vertical velocity and expected the vertical impulses to be similar.

During bipedal walking and running in humans, birds and macaques, the vectors of the ground reaction force point from the center of pressure (CoP) towards a point in the vicinity of the CoM (Andrada et al., 2014; Blickhan et al., 2018; Maus et al., 2010; Vielemeyer et al., 2021). The concept of the virtual pivot point (VPP) transfers the naval stability concept of a metacenter to bipedal locomotion (Fig. 1C,D). As during bipedal walking, the VPP is located above the CoM and the torques of the ground reaction forces with respect to the CoM seem to stabilize the system similar to a pendulum because of its suspension at the pivot point. With the transition to running, the distance between the VPP and CoM vanishes or even becomes negative (Vielemeyer et al., 2021). This was also observed during grounded running and aerial running in macaques (Blickhan et al., 2018). When walking up and down a step, the VPP is still observed but shifted with respect to the CoM (Vielemeyer et al., 2021). With the differential function of the legs during skipping, a shift of the VPP would indicate a shift in control.

Such differences should be accompanied by differences in leg function. The focus on global leg properties described by a compliant telescope unloads the investigation from considerations on joint angles and joint torques. It requires as kinematic properties leg length and the leg angle (Fig. 1E,F). The use of a lumped parameter model (spring–damper; Fig. 1E) to describe the dynamic force–length properties facilitates comparisons among species with deviations in leg design (birds: Andrada et al., 2013a; humans: Andrada et al., 2013b, 2016) and comparisons with results from numerical modelling (Andrada et al., 2014; Blickhan, 1989; Blickhan et al., 2015; Drama and Badri-Spröwitz, 2020). In the investigation on walking, grounded running and aerial running in macaques (Blickhan et al., 2018), compliant legs and deviations from pure energetically conservative, quasi-elastic operation were observed. These deviations were approximated by a damper in parallel to the spring where the damper should turn into a motor in case of leg lengthening. Leg stiffness did not differ between grounded and aerial running (Blickhan et al., 2018), but the contribution of the parallel damper shifted from a damper absorbing energy to a motor generating work. Some bird species prefer to skip despite having compliant legs (Verstappen and Aerts, 2000; Alexander, 2004). We expected that the macaques also use much more compliant legs than humans during skipping and a differential distribution of axial work and damping for the trailing and leading leg, respectively. The different operation of the legs should also affect the roll over the feet, i.e. the position of the CoP.

The reduction of a leg to an axial telescope ignores the tangential forces perpendicular to the telescope and the generated moments (Fig. 1C,D). These components were expected to be high for the ‘effective leg’ (Fig. 1C), the leg from the CoP to the hip, as it counteracts torques developed by the pitched trunk and its role might change in the trailing and leading phase. Torques developed by the ‘virtual leg’, connecting the CoP to the CoM, rotate the whole system. A VPP located away from the CoM indicates such torques. However, it does not inform about the net rotational impulse perpendicular to the sagittal plane generated by the torques. In regular, symmetrical gaits, the rotational impulse should be low for each step to avoid tipping over and to minimize corrections otherwise necessary from step to step. During skipping, the rotational impulse might differ during the trailing and leading phase in order to facilitate a secure landing and to redirect the impulse for take off. Nevertheless, the rotational impulses generated in the trailing and leading phase were expected to compensate each other during a stride.

The description of both the effective and the virtual leg is also relevant when comparing with numerical models. The virtual leg is used in lumped parameter models such as the spring-loaded inverted pendulum (SLIP) and subsumes the relative movement between the trunk and the effective legs (e.g. Blickhan, 1989; Andrada et al., 2013a, 2016, 2020). The values for the effective leg are relevant in models including a heavy trunk (Andrada et al., 2014; Blickhan et al., 2015; Drama and Badri-Spröwitz, 2019; Maus et al., 2010).

External forces and impulses accelerate the CoM. The energetics of the CoM, especially the phase between the changes in potential and kinetic energy as quantified in parameters such as recovery (Cavagna et al., 1977) and congruity (Ahn et al., 2004), informs us whether the gait can be considered dynamically as a walk (out of phase, stiff inverted pendulum) or a run (in phase, SLIP; Ogihara et al., 2018; Blickhan et al., 2018). The double support typical for skipping does not guarantee a walking-like step or a classification of the stride as being intermediate between walking and running. The macaques use grounded running, i.e. a running gait despite having two double supports and no aerial phase. Because of their compliant legs, reduced bouncing was expected. Therefore, the external mechanical cost of transport (CoT), determined by the fluctuations of the mechanical energy of the CoM, was expected to be less than in humans but higher when negotiating the hurdles.

The present study aimed to clarify the differential leg and trunk operation during skipping in bipedal macaques by analyzing ground reaction forces, the global properties of the effective and virtual leg, the location of the VPP, and the energetics of the CoM.

## MATERIALS AND METHODS

Most variables and their abbreviations and definitions are presented in Table 1 and explained in simple graphics in Fig. 1. In order to facilitate comparison with data from other primates and birds and with numerical calculations, we used dimensionless formulations (Hof and Zijlstra, 1997; Pinzone et al., 2016). We used for normalization the lengths given for the subjects below. More details of the methods largely repeated here for convenience are published in Ogihara et al. (2018) and Blickhan et al. (2018).

**Table 1.**
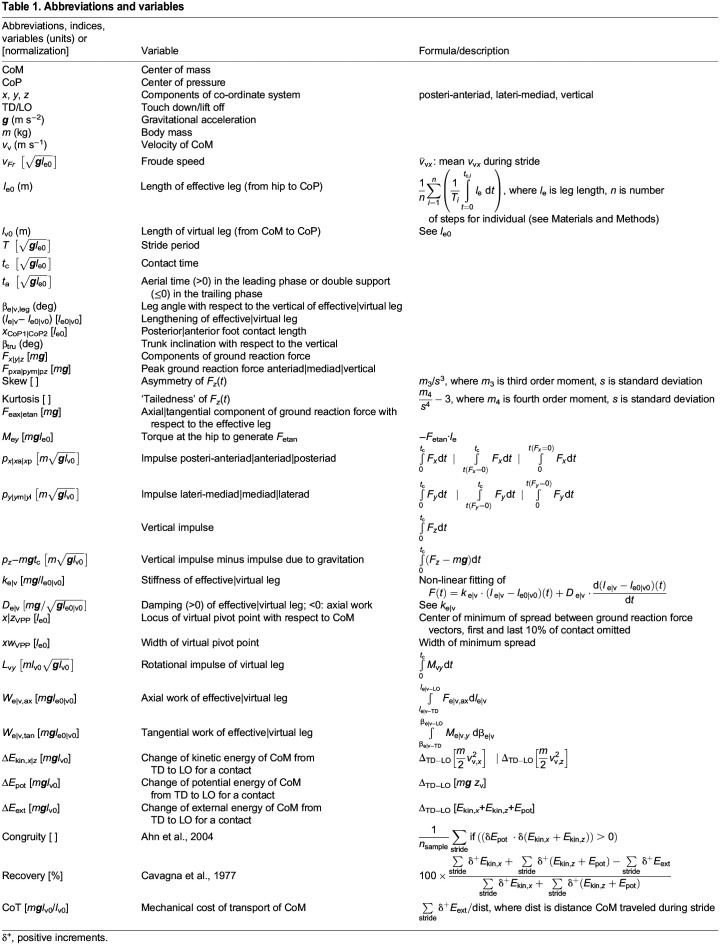
Abbreviations and variables

### Subjects

The macaques performed at the Suo Monkey Performance Association (Kumamoto, Japan). The three adult, male macaques (Ku, Po and Fu, age 15, 13 and 12 years; mass 8.64, 8.81 and 8.79 kg) had been trained for bipedal walking and performances since the age of about 1 year. The grand means (number of steps: 34, 2, 42) of leg lengths during the stance phases observed during grounded running and running were 0.384, 0.319 and 0.397 m for the effective leg, *l*_e0_, and 0.518, 0.450 and 0.591 m for the virtual leg, *l*_v0_, respectively. The reduced sample and the resulting shortening (effective leg: −8%; virtual leg: 4%) as compared with Ogihara et al. (2018) avoided potential bias due to the inclusion of asymmetrical gaits.

### Setup

The macaques run across a flat wooden track (length: 5 m) with two embedded force plates (0.4 m×0.6 m). During hurdling, two hurdles (height: 0.1 m) were placed at the beginning and the end of the two force plates (0.81 m apart; Fig. 1A). While the macaques crossed the track kinematics (10 s), ground reaction forces for two consecutive steps were captured with an eight-camera infrared motion capture system (Oqus 3+, Qualisys, Göteborg, Sweden) and the force plates (EPF-S-1.5KNSA13; Kyowa Dengyo, Tokyo), respectively, at a rate of 200 Hz.

### Procedure

An individual coach and caregiver guided the macaques across the track with a slack leash. Reflective markers (14 mm diameter, Vicon) were attached onto Velcro straps with double-sided tape. Macaques did not tolerate markers on the arms and head (Fig. 1B). A total of 15 markers were placed at the acromion (2), sternum xiphoid (1), tenth thoracic vertebra (1), anterior superior iliac spine (2), sacrum (1), greater trochanter (2), lateral epicondyle (2), lateral malleolus (2) and fifth metatarsal head (2). Joint centers of the knee, the ankle and the metatarsals were calculated as half the distance between medial markers placed in addition to the lateral markers during posing on the animal and during the trials by projecting from the lateral markers perpendicular to the main plane of movement of the knee. The location of the trochanter head was estimated by a similar projection from the greater trochanter marker with the distance between the marker and trochanter head obtained from cadaver measurements (Ogihara et al., 2009).

### Ethical statement

The experiments were approved by the Animal Welfare and Animal Care Committee, Primate Research Institute, Kyoto University. All institutional guidelines were followed for this study. By rewards, the macaques were easily motivated to walk bipedally. They were used to jumping across high hurdles. Speed was freely selected and experiments were stopped as soon as signs of unwillingness surfaced.

### Data evaluation

**Fig. 2.**
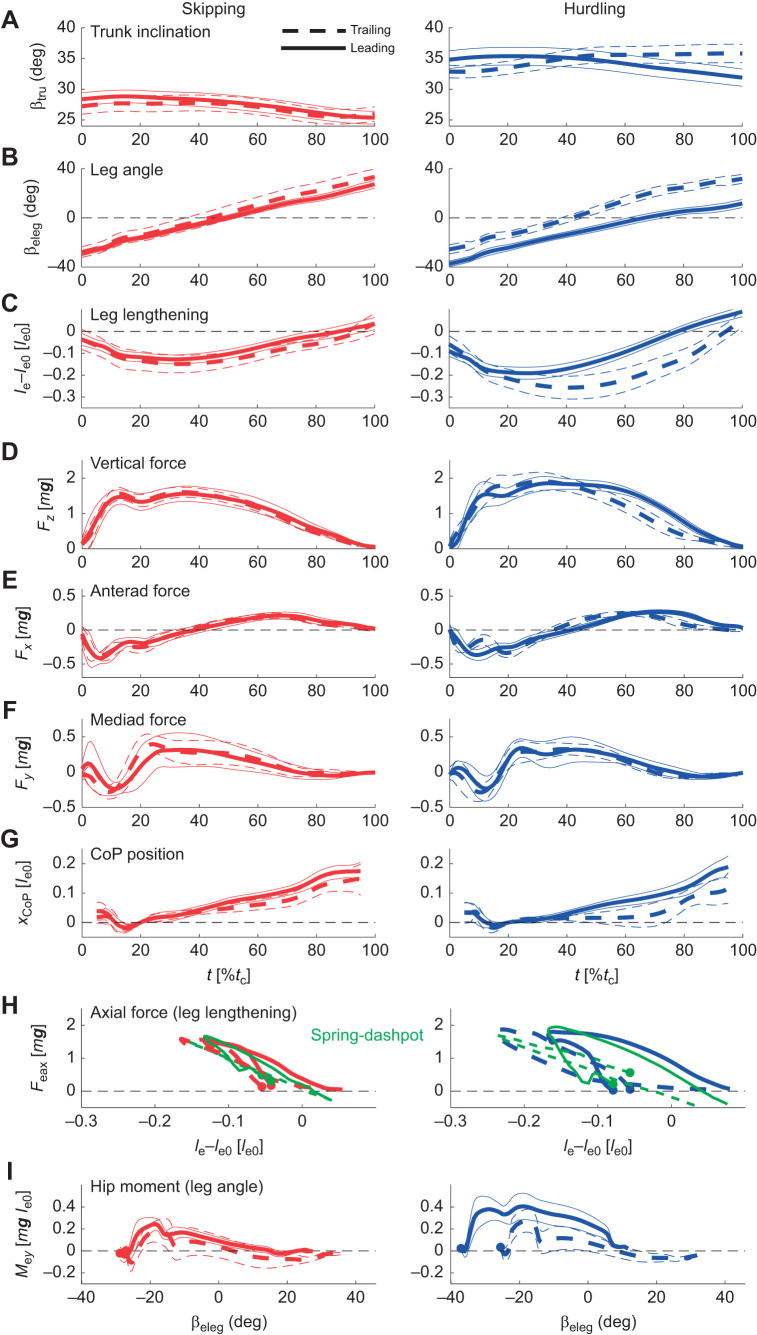
**Global kinematic and dynamic parameters.** Mean (bold lines) and s.d. (thin lines) of time courses of global properties during skipping (red) and hurdling (blue) in the trailing (dashed line) and the leading leg (solid line). A–G show time, *t*, normalized to contact time, *t*_c_. (A) Trunk pitch, β_tru_ (±s.e.m.). (B) Leg angle, β_eleg_. (C) Leg lengthening, *l*_e_−*l*_e0_. (D–F) Craniad, *F_z_*, anteriad, *F_x_*, and mediad, *F_y_*, components of ground reaction force. (G) Anteriad component of center of pressure, *x*_CoP_. *x*_CoP_ at 20%, *t*_c_ set to 0 and the first and last 5% were omitted. (H) Axial force (leg lengthening) loops, *F*_eax_(*l*_e_−*l*_e0_). For variance, see tracings in Fig. S1. Green dashed lines: fittings based on spring dashpot (Voigt) model (Fig. 1). Filled circles: touch down. (I) Tangential torque (leg angle) loops, *M*_e*y*_(β_eleg_); filled circles: touch down. Mean (±s.d.) contact times: *t*_c,skip,trail_=0.225±0.036 s; *t*_c,skip,lead_=0.214±0.039 s; *t*_c,hurd,trail_=0.240±0.033 s; *t*_c,hurd,lead_=0.210±0.016 s.

**Fig. 3.**
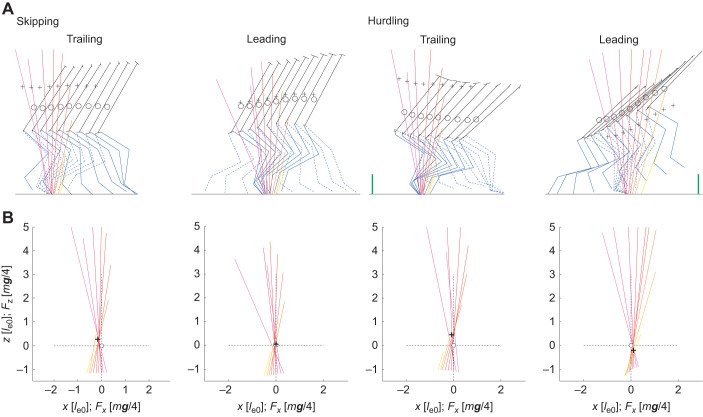
**Kinematics and dynamics during skipping and hurdling.** (A,B) Stick figures and VPP plot. In the stick figures (A), two consecutive steps of a single trial are depicted, i.e. stick figures are overlapping (macaque: Fu). Circle, CoM; cross, VPP. Solid black line, trunk; solid blue line, right leg; dashed blue line, left leg (note: different legs are used as trailing and leading legs in the two trials for skipping and hurdling); green lines within the two graphs during hurdling indicate the hurdles (to scale). The VPP graphs (B) are for the trials depicted in A. Contact times are divided into 8 segments. The color of the force vectors shifts with time from magenta to orange. Forces: 2 *m**g*** m^−1^. From left to right−speed: 2.2, 2.2, 1.7, 1.7, m s^−1^; *l*_e0_: 396, 392, 711, 826 mm; floor lines: 647, 889, 711, 826 mm; *t*_c_: 0.21, 0.22, 0.295, 0.255 s; *t*_da_: −0.015, 0.025, −0.035, 0.185 s; *x*_VPP_: −59, −1, −29, 47 mm; *z*_VPP_: −107, 24, 173, −87 mm.

The CoM of the trunk has been located on the line connecting mid hip joint (midpoint of the left and right joint centers) and mid shoulder. Based on the location of the markers, the position of the segmental CoM and the instant position of the CoM of the individual were obtained using morphometric data (Ogihara et al., 2011). Within a presentation of the ground reaction forces (Figs 1D,G, 2D–F) with respect to the instantaneous CoM (CoM-fixed coordinate system), the VPP was calculated as the center of the waist (minimum horizontal width) established by the crossing of the extended ground reaction force vectors (first and last 10% of contact time omitted; Figs 1C and 3; Blickhan et al., 2018). The CoP was registered by the force platform in combination with the markers at the lateral malleolus and fifth metatarsal head.

In the present study, we focused on skipping and hurdling. Skipping was identified by a double support phase followed by an aerial phase (Fig. 1A). Both phases were decoded via the variable aerial phase, *t*_da_, with *t*_da_≤0 indicating double support and *t*_da_>0 indicating flight. The step and the leg in advance of the double support are termed ‘trailing’, and those in advance of the aerial phase are termed ‘leading’. In order to facilitate statistics as well as the analysis of the motion of the trunk segments, only sequences where a complete dataset was available for both steps and no stumbling and distraction was observed were selected for further analysis. This selection resulted in a sample of 18 (Ku: 2, Fu: 8, Po: 8) strides for skipping and 31 (Ku: 4, Fu: 22, Po: 5) strides for hurdling. Global parameters where investigated during stance. The description of CoM data included a stride.

The effective leg reaches from the CoP to the greater trochanter, and the virtual leg reaches from the CoP to the CoM (Fig. 1C).

Leg stiffness, *k*, and damping, *D*, were calculated by fitting a parallel arrangement of a linear spring and a damper to the individual axial force–leg length data [*F*_eax_(*l*), Table 1; Figs 1D,J and 2H] and we used a dimensionless formulation (Blickhan et al., 2018). The axial leg properties were complemented by tangential properties derived from leg torque–leg angle data [*M*_e*y*_(β_eleg_)=*l*_e_*F*_etan_(β_eleg_), Table 1; Figs 1K and 2I]. Kinetic energy of the CoM, 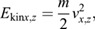 and potential energy, *E*_pot_=*m**g**z*, were calculated by integration of the accelerations, *a_x_*_,*z*_, obtained from the ground reaction forces, *a_x_=F_x_*/*m*, and vertical ground reaction force, 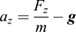 using displacements and velocities of the CoM obtained from kinematics at the boundaries of the two contacts (*t*_TD,trail_=0,*t*_LO,lead_; see Equations 1--3 in Supplementary Materials and Methods).

Congruity (Ahn et al., 2004) specifies the fraction within a stride in which kinetic and potential energy are in phase (Table 1). The range from 0 to 0.5 is accepted as walking (*E*_pot_ and *E*_kin_ largely out of phase) and the range from 0.5 to 1 as a bouncing gate (*E*_pot_ and *E*_kin_ largely in phase). Recovery is low for bouncing gaits (Cavagna et al., 1977).

The combined influence of leg (trailing versus leading), Froude speed as a covariant, and of the individuals was tested with a general linear model (hierarchic-type I with repetitions; Bonferroni correction *f*=141, IBM^®^SPSS^®^, Armonk, NY, USA). The repetition refers to the steps of the leading and the trailing leg within the same stride (Table 2; Table S1). This was complemented by univariate comparisons between skipping and hurdling considering the covariant Froude speed and the factor subject (Table 2; Table S2).

**Table 2.**
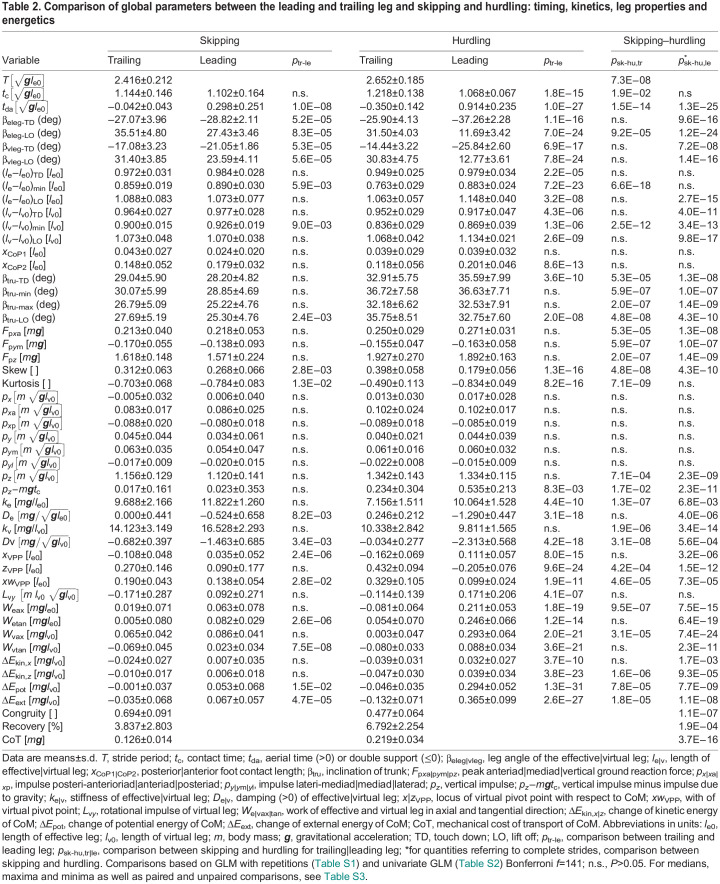
Comparison of global parameters between the leading and trailing leg and skipping and hurdling: timing, kinetics, leg properties and energetics

Depending on the normality of distribution (Lilliefors test), parametric (*t*-test, unpaired *t*-test) or non-parametric tests (Wilcoxon sign-rank test, Wilcoxon) were performed (Table S3).

Custom software was written in MATLAB 14 (MathWorks, Natick, MA, USA).

## RESULTS

### Global kinematics

**Fig. 4.**
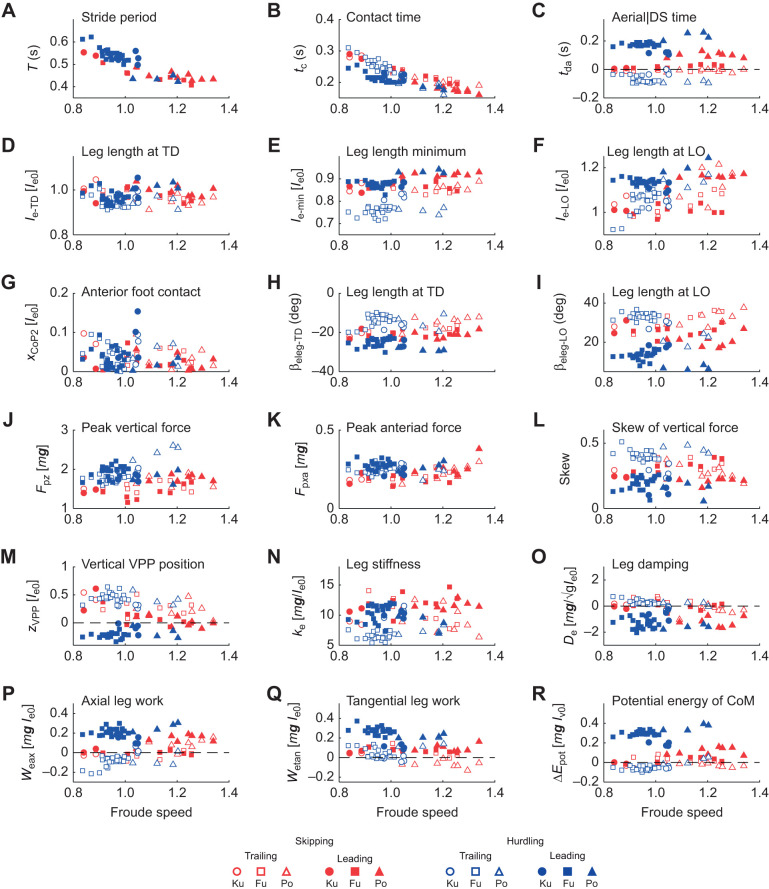
**Dependence of kinematic, dynamic and energetic stance parameters on Froude speed.** Red, skipping; blue, hurdling; open symbols, trailing leg; filled symbols, leading leg. (A–C) Periods: (A) stride period, *T*; (B) contact time, *t*_c_; and (C) aerial time, *t*_da_ (>0 flight; ≤0 double support). (D–F) Length of the effective leg: (D) at touch down, *l*_e−TD_; (E) at minimum length, *l*_e−min_; and (F) at lift off *l*_e−LO_. (G) Length of CoP progression after 20% total length, *x*_CoP2_. (H,I) Angle between leg and vertical: (H) at touch down, β_eleg-TD_; (I) at lift off, β_eleg-LO_. (J,K) Amplitude of the ground reaction force: (J) vertical force, *F*_p*z*_; (K) anterior force, *F*_p*x*a_. (L) Skew of vertical force. (M) Elevation of the VPP above the CoM, *z*_VPP_. (N) Stiffness of the effective leg, *k*_e_. (O) Damping of the effective leg, *D*_e_. (P–R) Work and energy: (P) axial work of the leg, *W*_eax_; (Q) tangential work, *W*_etan_; (R) change in potential energy of the CoM, Δ*E*_pot_. For additional information, see Fig. 5.

**Fig. 5.**
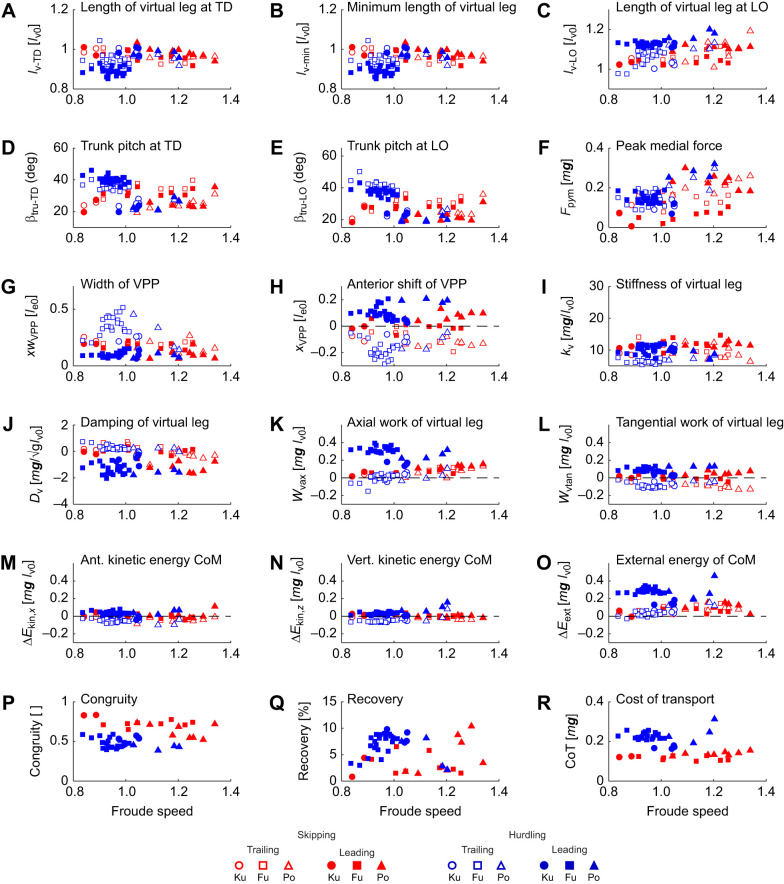
**Dependence of kinematic, dynamic and energetic stance parameters on Froude speed.** Red, skipping; blue, hurdling; open symbols trailing leg; filled symbols: leading leg. (A–C) Lengths of the virtual leg from CoM to CoP: (A) at touch down, *l*_v−TD_; (B) at minimum length, *l*_v−min_; and (C) at lift off, *l*_v−LO_. (D,E) Trunk angle with respect to the vertical: (D) at touch down, β_tru-TD_; (E) at lift off, β_tru-LO_. (F) Amplitude of the medial ground reaction force, *F_y_*. (G) Width of the VPP, *xw*_VPP_. (H) Anterior shift of the VPP, *x*_VPP_. (I) Stiffness of the virtual leg, *k*_v_. (J) Damping of the virtual leg, *D*_v_. (K,L) Work of the virtual leg: (K) axial work, *W*_vax_; (L) tangential work, *W*_vtan_. (M,N) Changes in kinetic energy of the CoM: (M) anterior, Δ*E*_kin,*x*_; (N) vertical, Δ*E*_kin,*z*_. (O) Changes in external energy, Δ*E*_tot_. (P) Congruity[ ], dimensionless. (Q) Recovery. (R) CoT.

The macaques used different leg angles in the trailing and leading period. Stride period, *T*, decreased with speed with individual variance (Fig. 4A; Table S1). Contact times, *t*_c_, were mostly shorter in the trailing leg than in the leading leg during hurdling (Fig. 4B; Table 2). The aerial time, *t*_da_, was used for classification. During skipping, an aerial phase (*t*_da_>0 s) follows a double support phase (*t*_da_≤0 s). The double support phase during skipping was always rather short (≥−0.02 s; Fig. 4C). Trunk posture, β_tru_, showed a high interindividual variance (Figs 2A, 5D,E, Fig. S1A; Table S1). In most cases, β_tru_ decreased during stance. One subject (Fu) righted itself in the trailing phase during hurdling. At lift off, β_tru-LO_ was higher in the leading than in the trailing phase (Table 2), i.e. the subject was more erect. The leg lengthened during stance [(*l*_e_−*l*_e0_)_TD_>(*l*_e_−*l*_e0_)_LO_; Figs 2C and 4D,F; Fig. S1C]. This was most pronounced in the leading leg while hurdling. There (*l*_e_−*l*_e0_)_TD,LO_ strongly differed in the trailing and leading phase (Table 2). Leg compression was most pronounced in the trailing leg while hurdling and the maximum compression was shifted towards midstance (Fig. 2C, Table 2; Fig. S1C). During hurdling, leg rotation, β_eleg_, was shifted towards a flatter leg angle at touch down and a steeper angle at take off in the leading leg (Figs 2B and 4H,I; Fig. S1B; β_eleg-TD,LO_, Table 2).

### Forces, CoP and VPP

The time courses of the ground reaction forces, *F_x_*_,*y*,*z*_(*t*), were rather similar in the trailing and leading leg and during skipping and while crossing the hurdles (Fig. 2D–F). Nevertheless, skew of the vertical force, *F_z_*, was higher in the trailing than in the leading leg and the inverse was true for kurtosis (Figs 2D and 4L; Fig. S1D; Table 2). The higher peak vertical force, *F*_p*z*_, while jumping the hurdles were accompanied by increased propulsive forces, *F*_p*x*a_ (Fig. 4J and Table 2). However, they did not differ in the leading and trailing leg (Table 2). The peak medial force, *F*_p*y*m_, varied interindividually (Fig. 5F; Fig. S1F, Table S1). Macaque Fu pulled inside with the trailing and with the leading leg (Fig. S1F). The progress of the CoP, *x*_CoP_, was reduced in the trailing leg during hurdling (Figs 2G and 4G, Table 2; Fig. S1G, Table S2), which was most pronounced for a youngest subject (Fu). The VPP (Fig. 3, Table 2) was located posterior (*x*_VPP_<0) and above (*z*_VPP_>0) the CoM for the trailing leg during both skipping and hurdling (Table 2). In the leading phase, it was more focused (width of the pivot point, *xw*_VPP_; Table 2) and shifted towards the CoM (skipping) and anterior (*x*_VPP_>0) below (*z*_VPP_<0) the CoM during hurdling (Figs 2B and 4M). The horizontal shift of the VPP, *x*_VPP_, and also the vertical shift, *z*_VPP_, during hurdling strongly differed in the trailing and leading period (Table 2).

### Leg stiffness, leg torque and leg work

During hurdling, stiffness, *k*_e_, of the effective leg in the trailing phase was below the stiffness in the leading phase (significant for hurdling; Fig. 4N, Table 2). The force–length loops indicate energy absorption in the trailing leg during hurdling and positive axial work, *W*_eax_, in the leading leg (Figs 2H and 4P, Table 2; Table S1). In the oldest subject (Ku) with its longer training history, these differences were less pronounced (Fig. 4P; Fig. S1H). The moment angle loops were positive in the leading leg, being higher than the values observed in the trailing leg during both skipping and hurdling (*W*_etan_; Figs 2I and 4Q; Fig. S1I; Table 2). The tangential work, *W*_etan_, tended to decrease with Froude speed. The differences in potential energy of the CoM between lift off and touch down, Δ*E*_pot_, indicated a slight lowering of the CoM in the trailing phase and a lift especially during hurdling (Fig. 4, Table 2). Similarly, during hurdling, the differences in vertical kinetic energy, Δ*E*_kin,*z*_, indicated a reduction in the trailing phase and an increase in the leading phase (Fig. 5, Table 2). Congruity was above 50% for skipping but about 50% for hurdling. Recovery was always below 20% during the stride (Table 2).

## DISCUSSION

### Skipping

Walking, grounded and aerial running are lateral-symmetrical. In contrast, skipping and hurdling are by definition lateral-asymmetrical. A differential operation of the leading and trailing legs was observed.

The aerial phase was not due to an elevated impulse generated at the leading leg but due to the double support phase. A difference between trailing and leading with respect to the peak ground reaction force, *F*_p*x*,*y*,*z*_, was not observed (Table 1). The impulse, *p_x_*_,*y*,*z*_, generated by each leg did not differ even when considering for the vertical component the adverse action of gravity (−*m**g**t*_c_). The more platykurtic form of the vertical force component, *F_z_*(*t*), of the leading leg was compensated by its reduced contact time, *t*_c_. Positive skewness was reduced in the leading leg as compared with the trailing leg. This reduces the combined impulse during double support. Nevertheless, the impulse generated by the combined action of both legs during the stride was sufficient to generate the short aerial phase after lift off of the leading leg. The time course of the vertical velocity (Fig. S2) shows that the double support reduces the drop of the velocity. The double support was necessary to generate sufficient total impulse for the short aerial phase.

The differences in timing of the legs were accompanied by differences in leg operation with respect to leg kinematics and leg compliance. The slightly lower minimum of the trailing leg length, (*l*_e_−*l*_e0_)­_min_, indicates a higher leg compression (Fig. 2C, Table 2). A similar peak force at higher leg compression indicates a reduced leg stiffness, *k*_e_, for the trailing leg. This is substantiated especially for hurdling by the fittings of the spring-damper models (Figs 2H, 4N, Table 2). In other words, the same peak force within a reduced contact time was generated in the leading leg with increased leg stiffness.

Differences in kinematics resulted in differential energetics. The trailing leg appeared to operate quasi-elastic at the global level. In contrast, the leading leg lengthened and generated work (*W*_eax_; Table 2). The leg angle at touch down, β_eleg-TD_, was flatter in the leading as compared with the trailing phase, and it was steeper in the leading leg at lift off, β_eleg-LO_ (Table 2). The flat leg angle at lift off, β_eleg-LO_, of the trailing leg facilitated the generation of the double support. It also resulted in a slight lowering of the CoM in the trailing phase followed by a lift in the leading phase (Δ*E*_pot_; Table 2). The double support provided the impulse to stop the falling of the CoM in the trailing phase and secured sufficient impulse to lift the CoM in the leading phase. Despite the higher vertical impulse, *p_z_*, as compared with the horizontal, *p_x_*, the mixed terms resulted in higher fluctuations of the horizontal velocity. The fluctuations in kinetic energy were less than those of the potential energy (Fig. S2). The changes in external energy of the CoM, Δ*E*_ext_, were dominated by the contributions of the potential energy, Δ*E*_pot_. The dimensionless values of the latter correspond to the dimensionless changes in lift, which were 5% *l*_v0_ or about 2.5 cm. The macaques had a smooth ride during skipping. Nevertheless, with ca. 70% congruity and ca. 4% recovery, skipping in macaques was classified as a bouncing gait. The virtual leg did axial work, *W*_vax_ (Table 2), in agreement with the increase of total translational energy of the CoM. To assure falling on their arms, the net rotational impulses, *L*_v*y*_, generated by the ground reaction force with respect to the CoM were clockwise in the trailing phase and then reversed in the leading phase (n.s., Table 2). This is supported by the placement of the virtual pivot point behind, *x*_VPP_, and above, *z*_VPP_, the CoM in the trailing leg and very close to the CoM in the leading phase. During the latter, the ground reaction forces focused more precisely (*xw*_VPP_). The trunk was slightly more erect before lift off, β_tru-LO_, of the leading leg. The leading leg produced tangential work, *W*_etan_, to compensate for the rotational impulse (Table 2). The hip placement combined with the macaque's posture enforced tangential work of the effective leg during retraction. Unfortunately, there remained an imbalance in our trials: the tangential work produced by the virtual leading leg, *W*_vtan_, was less than the absorption in the trailing phase (Table 2). This as well as the unbalanced rotational impulse, *L*_v*y*_, was also reflected in the asymmetric placement of the virtual pivot point considering the two steps.

### Hurdling and differences to skipping

The differences between the kinetic parameters describing the trailing and leading steps were much more accentuated during hurdling (Table 2; Tables S2 and S3).

Surprisingly, peak ground reaction force, *F*_p*x*,*y*,*z*_, and the impulses, *p_x_*_,*y*,*z*_, during hurdling did not differ between the legs despite their enhanced value with respect to skipping (Table 2). However, the vertical impulse after considering gravity clearly differed between the legs for hurdling (Table 2). In the leading leg, the contact time, *t*_c_, was not shorter than during skipping, despite the slight lengthening of the contact during the trailing phase, and a lengthening in the stride period, *T* (Table 2). During skipping, the double support was only marginal but it was largely enhanced during hurdling. It was this enhanced double support that provided the impulse to clear the hurdles. During hurdling as compared with skipping, differences in form of the time courses of the vertical component of the ground reaction force of the trailing and leading leg were much more accentuated: during hurdling, left skewness was enhanced in the trailing leg and reduced in the leading leg, and kurtosis (excess) was reduced in the trailing leg and enhanced in the leading leg as compared with skipping (Table 2). The skewness indicates enhanced landing impacts after the flight phase. The leading leg was stiffer, *k*_e_, than the trailing leg, i.e. during skipping, a lower compression (*l*_e_−*l*_e0_)_­min_ within a reduced contact time resulted in similar peak forces (Table 2). As compared with skipping, in hurdling, the trailing leg was even more compliant (Table 2). A strong extension of the leading leg was observed at lift off, (*l*_e_−*l*_e0_)­_LO_, resulting in a high lift of the CoM during contact (Δ*E*_pot_, Table 2). This was amplified by a rather steep leg angle at lift off (β_eleg-LO_; Table 2). The leading leg was placed under a flat leg angle, β_eleg-TD_, and operated in a much more asymmetric mode as compared with the trailing leg. This change of the leg angle supported the longer roll off distance of the foot in the leading phase (*x*_CoP2_; Table 2). The leading leg produced axial work (*W*_eax,hip_; Tables 1 and 2). This is expressed in the negative damping, *D*_e_, parallel to the leg spring (Table 2). In the trailing leg, the damper absorbed energy. The axial work of the virtual leg, *W*_vax_, was also concentrated on the leading leg (Table 2). The differences of the positions of the virtual pivot point, *x*,*z*_VPP_, were more accentuated than during skipping, indicating a more walking-like trailing step and running-like leading step (Table 2). As during skipping, tangential work of the leg, *W*_etan_, was observed in both legs with clearly higher values in the leading phase. As in skipping, the imbalance in tangential work within the stride was reduced in the virtual leg (*W*_vtan_) as also indicated by the differences of the generated rotational impulse (*L*_v*y*_; Table 2). The considerable tangential work, *W*_etan_, in the leading leg was counteracted by the erect trunk (β­_tru_; Table 2). The posterior placement of the hip, in combination with the requirement to focus the forces to the CoM in preparation of the aerial phase, enforced tangential work of the leg. The leg moment was counteracted by the movement of the trunk. Remarkably, the increased double support and the continued vertical acceleration of the CoM (Fig. 5) resulted in a congruity of about 50%, which is reduced as compared with that during skipping and is conceived as being the border between walking and running (e.g. Andrada et al., 2013b).

By placing the hurdles before and after the two force plates, we induced skipping across hurdles. Without hurdles, skipping was the gait preferred by the macaques at higher Froude speeds (Ogihara et al., 2018). Skipping seemed to be convenient. This may be why the macaques rarely tried to run regularly. The fact that the macaques crossed the hurdles with ease shows that they were able to generate higher flight phases using a similar bipedal rhythm. During skipping, there were differences with respect to the operation of the trailing and leading leg. However, these differences represented only a very minor deviation from the standard mode of operation. As indicated above, the use of a double support phase, i.e. a rhythmical parameter, seemed to be essential to generate the short flight. The differential leg parameters were useful to cope with the consequences. During hurdling, these differences were largely exaggerated. Here, both kinematic and kinetic properties of the legs and its joints, as well as the role of the trunk strongly supported the different function of the trailing and leading leg. Two of the subjects had a preferred side (trailing leg for each individual: left|right, Ku: 6|0; Fu: 11|19; Po: 0|14).

### Comparison of skipping in human and macaque

Human parameters differ in some respect from those observed in macaques and the more accentuated observations during hurdling. The leg angle, β_eleg_, with respect to the horizontal axis was smaller at touch down of the trailing leg and at lift off of the leading leg during skipping (macaque skip|hurdle|human skip, β_eleg,trail-TD,TO_: 62.9,125.5|64.1,121.5|82.1,122.2 deg; β_eleg,lead-TD,TO_: 61.2,117.4|52.7,101.7|57.4,103.1 deg; Müller and Andrada, 2018). Leg lengthening, Δ*l*_e_=*l*_eLO_−*l*_eTD_, is more expressed in the macaque (Δ*l*_etrail_: 0.11|0.11|−0.01 [*l*_e0_]; Δ*l*_elead_: 0.09|0.17|0.025 [*l*_e0_]; Müller and Andrada, 2018). Lift of the CoM, Δ*z*_CoM_ [*l*_e0_]=Δ*E*_pot_ [*m**g**l*_e0_], is less for skipping in macaques but higher for hurdling as compared with human skippers (Δ*z*_CoM,trail_: −0.05|−0.16|0.1 [*l*_e0_]; Δ*z*_CoM,lead_: 0.06|0.22|0.09 [*l*_e0_]; Müller and Andrada, 2018) but in all cases the CoM is lowered in the trailing and lifted in the leading phase. The macaques used in general similar leg movements but with lower leg stiffness (see below).

As in macaques, unilateral skipping amplitudes of the vertical component of the ground reaction force (*F*_p*z*,trail_=2.28 [*m**g***], *F*_p*z*,lead_=2.14 [*m**g***]) and its impulse (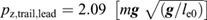) did not differ significantly between the leading and the trailing leg in humans (after Fiers et al., 2013). However, the amplitudes by far exceed even the values observed in macaques during hurdling and so do the anteriad impulses (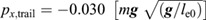; 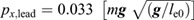). In both species, the trailing leg is decelerating and the leading leg is accelerating. However, the decelerations and accelerations as related to the vertical impulse in the macaque were less than 20% of the contributions during human skipping. During hurdling in macaques, both legs were accelerating and the ratios between horizontal and vertical impulses almost reached the human values. The lower oscillation of the horizontal energy in the macaque during skipping seems to be a matter of convenience. There was net acceleration in our trials during hurdling; the track allowed about three strides. The human subjects preferred to locomote on a treadmill at about the same Froude speed (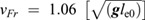) but with a considerably shorter stride duration (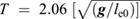). Correspondingly, the contact times were shorter (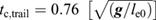; 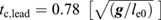). Human bipedal gallopers used a higher leg stiffness as compared with macaques. This was also confirmed in a recent study where kinematic parameters were used to estimate leg stiffness during unilateral skipping or galloping (Pequera et al., 2021). In that study, the stiffness of the trailing leg by far exceeded the values obtained for the leading leg and the values obtained in our study for the macaques (from regression *v_Fr_*=1: *k*_trail_=46.4 [*m**g***/*l*_e0_]; *k*_lead_=23.8 [*m**g***/*l*_e0_]; Pequera et al., 2021). In our study of human unilateral skipping, stiffness of the leading leg was enhanced as in the macaque (*v_Fr_*≈1: *k*_trail_=34.9 [*m**g***/*l*_e0_]; *k*_lead_=44.1 [*m**g***/*l*_e0_]; Müller and Andrada, 2018). The macaques skipped with much more compliant legs.

For the trailing leg, the direction of the ground reaction force as quantified by *z*_VPP_ during skipping resembled the values found for the macaques during running (0.20 [*l*_e0_]), whereas during hurdling, they resembled the values found during grounded running (0.38 [*l*_e0_]; Blickhan et al., 2018). Both are within the range found during human walking (Maus et al., 2010; Vielemeyer et al., 2019). In the leading leg, the values move closer to the CoM and for hurdling even below the CoM. This resembles human running (Maus et al., 2010). At high speeds during human running, the values are below the CoM (Drama and Badri-Spröwitz, 2020). Simulations demonstrate (Drama and Badri-Spröwitz, 2019) that the VPP in the vicinity of the CoM as found during slow human running facilitates exchange of energy between trunk and legs. For human walkers, the horizontal displacement of the VPP, *x*_VPP_, moves with increased trunk flexion posterior (Müller et al., 2017). In the macaques, the different location of the VPP in the leading and trailing leg correlated with the transmitted rotational impulse, *L*_v*y*_. However, the slight differences between the trailing and leading leg were not significant during skipping. Nevertheless, whole-body rotational impulse seems to be modified to provide secure landing in the trailing period and may also support take off in the leading phase. During hurdling, the influence of the extended double support may affect the location of the VPP. During human walking, *z*_VPP_ drops to zero during the double support and loses focus (Vielemeyer et al., 2021). This may help to adjust rotational moments and posture from step to step.

The energy of the CoM from touch down to lift off in humans indicates horizontal acceleration in the trailing leg and deceleration the leading leg (Δ*E*_kin,*x*,trail_=0.098 [*m**g**l*_v0_]; Δ*E*_kin,*x*,lead_=−0.084 [*m**g**l*_v0_]; Fiers et al., 2013), a vertical deceleration in the trailing and an acceleration in the leading leg (Δ*E*_kin,*z*,trail_=−0.044 [*m**g**l*_v0_]; Δ*E*_kin,*z*,lead_=0.037 [*m**g**l*_v0_]), and a lowering of the CoM in the trailing and a lift in the leading leg (Δ*E*_pot,trail_=−0.110 [*m**g**l*_v0_]; Δ*E*_pot,lead_=0.141 [*m**g**l*_v0_]). Such a pattern has also been documented in a trial in the pioneering study on bilateral skipping of Minetti (1998) (*v_Fr_*=0.84; Δ*E*_kin,*x*,trail_=0.078 [*m**g**l*_v0_]; Δ*E*_kin,*x*,lead_=−0.046 [*m**g**l*_v0_]; Δ*E*_kin,*z*,trail_=−0.040 [*m**g**l*_v0_]; Δ*E*_kin,*z*,lead_=0.032 [*m**g**l*_v0_]; Δ*E*_pot,trail_=−0.121 [*m**g**l*_v0_]; Δ*E*_pot,lead_=0.128 [*m**g**l*_v0_]), and in the early study of Caldwell and Whitall (1995) (*v_Fr_*≈1; Δ*E*_kin,trail_≈0.05 [*m**g**l*_v0_]; Δ*E*_kin,lead_≈−0.06 [*m**g**l*_v0_]; Δ*E*_pot,trail_≈−0.07 [*m**g**l*_v0_]; Δ*E*_pot,lead_≈0.1 [*m**g**l*_v0_]). This deviates from the pattern found in the macaque: in the macaque the horizontal and vertical kinetic energy decreased in the trailing leg and increased in the leading leg (Table 2). Human unilateral skippers also lower the CoM in the trailing phase to use a flatter leg angle of the leading leg to redirect the horizontal kinetic energy gained in the trailing phase to generate lift for flight. As in the macaques, according to the energetics of the CoM, skipping steps in humans were of the running type. There was no exchange between potential and kinetic energy or an inverted pendulum (compare with discussion in Fiers et al., 2013). In contrast to the recovery values for bilateral skipping (35–55%; Minetti, 1998), Pavei et al. (2015) documented recovery values for unilateral skipping close to running values (*v_Fr_*=1.01; recovery=21%). Recovery was even lower for the skipping and hurdling macaques. The congruity values for skipping macaques were rather similar to the values obtained in the macaques during grounded and aerial running (Ogihara et al., 2018). Unilateral skipping represents an intermediate gait between grounded and aerial running. (The extended double support during hurdling modified the energetics of CoM.) The external mechanical CoT, observed in the macaques during skipping and hurdling was of similar magnitude to the values observed during fast bilateral skipping in humans (0.08<CoT [*m**g***]<0.25; Minetti, 1998). For unilateral skipping (CoT≈0.1 [*m**g***], Caldwell and Whitall, 1995; 0.17 [*m**g***], Fiers et al., 2013), similar and higher values have been documented. During skipping, macaques avoided a bumpy ride.

In human locomotion, skipping is more expensive than running. If we assume this is also the case for the macaques then why did they prefer this gait? One reason could be stability. In the numerical simulation, the point of operation (β_vleg,trail-TD_=72.9 deg; β_vleg,lead-TD_=68.9 deg; *k*_v,trail_=14.1 [*m**g***/*l*_v0_]; *k*_vlead_=16.5 [*m**g***/*l*_v0_]) is close to but outside the self-stable region for a skipper with purely elastic legs (Andrada et al., 2016). However, this ignores the possibly stabilizing influence of the force generation (negative damping) parallel to the spring (*D*_v,trail_=−0.68 [*m**g***/(***g****l*_v0_)^1/2^]; *D*_v,lead_=−1.46 [*m**g***/(***g****l*_v0_)^1/2^]). The external mechanical cost of transport of the CoM, CoT, was less for grounded running (GR) and slightly less for running (R) but much higher for hurdling (mean±s.d. CoT_GR_=0.074±0.01 [*m**g***], *p*_GR,sk_=3*E*−7, *p*_GR,hu_=9*E*−10, *n*_GR_=38; CoT_R_=0.101±0.013 [*m**g***], *p*_R,sk_=1*E*−4, *p*_R,hu_=2*E*−11, *n*_GR_=46). We did not measure oxygen consumption. However, as in human locomotion, with respect to mechanics, skipping seems to be less convenient than symmetrical gaits. Despite bipedal training, our macaques prefer quadrupedal locomotion, possibly because of its reduced energetic cost (Nakatsukasa et al., 2004, 2006). During fast quadrupedal locomotion, they prefer a transverse gallop with a dominant hindlimb contribution (Kimura, 1992). Unilateral skipping or bipedal galloping represents a transverse gallop without forelimbs. Skipping may represent a preferred motor pattern for the bipedal macaques. Recent findings indicate that quadrupeds walk and trot with VPPs above the hip and the scapula (Andrada et al., 2023). It thus remains intriguing whether the differences in VPP heights depicted here between trailing and leading limbs hold for quadrupedal gallop, or whether they represent an adaptation to bipedal skipping.

### Conclusion

Based on recovery, skipping in macaques was classified as a running gait. The stepping pattern classed it as intermediate to grounded and aerial running. A slight shift in coordination between the left and right leg was sufficient to change gait. The shift in coordination was accompanied by a modification in the touch down and lift off angles of the leg in the leading with respect to trailing phase. Only an insignificant increase in leg stiffness and decrease in contact time were observed during the leading as compared with the trailing phase and there was no change in the vertical impulse. Despite the much lower leg stiffness in the macaque, this parallels human skipping. Nevertheless, the negative damping in the leading phase, along with additional tangential work and the shifted leg angles modified the time course of the ground reaction force. This alteration shifted the location of the VPP from grounded running-like for the trailing leg to running-like for the leading leg. These adjustments contributed to lifting the CoM in preparation of a short aerial phase. The accentuated dynamics observed while the macaques were skipping across hurdles and the low external COT indicated that skipping was not limited by the ability to generate forces but was selected by convenience, possibly facilitated by a co-ordination pattern adopted during quadrupedal locomotion.

## Supplementary Material

10.1242/jexbio.246675_sup1 Supplementary information
