# Supplementary material for: Skipping without and with hurdles in bipedal macaque: global mechanics
Source: J Exp Biol. 2024 Mar 28;227(7):jeb246675. doi: 10.1242/jeb.246675 (PMC11007588; doi:10.1242/jeb.246675)
Supplement: Supplementary information [file jexbio-227-246675-s1.pdf]

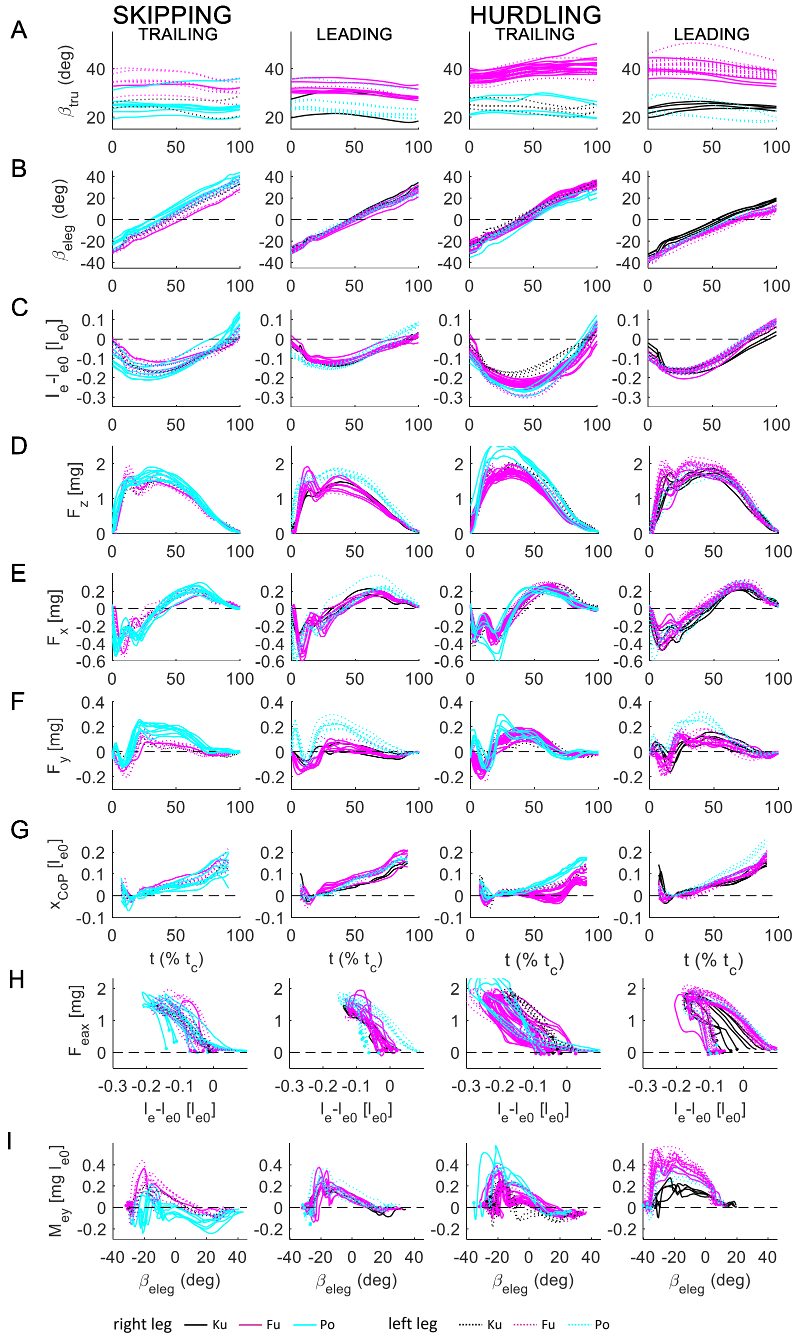

Fig. S1. Individual global properties during skipping and hurdling in the trailing and the leading leg. A-G) Time courses. t, time normalized to contact time, t_c. A) Trunk pitch, β_{tru}. B) Leg angle, β_{eleg}. C) Change of leg length, $(l_e - l_{e0})$ $l_{e0}$$^{-1}$. D-F) Craniad, F_z, anteriad, F_x, and mediad, F_y, components of ground reaction force. G) Anteriad omponent of center of pressure, x_{CoP}. x_{CoP} at 20% t_c set to 0 and the first and last 5% are omitted. H) Axial force length loops, $F_{eax}\left((l_e - l_{e0})l_{e0}{}^{-1}\right)$. Green dashed lines: fittings based on Voigt-model. Filled circles: touch down. I) Tangential moment angle loops, $M_{ey}(\beta_{eleg})$. Filled circles: touch down. Right leg: solid lines; left leg: dashed; macaques: black, Ku, magenta, Fu, cyan, Po.

Journal of Experimental Biology • Supplementary information

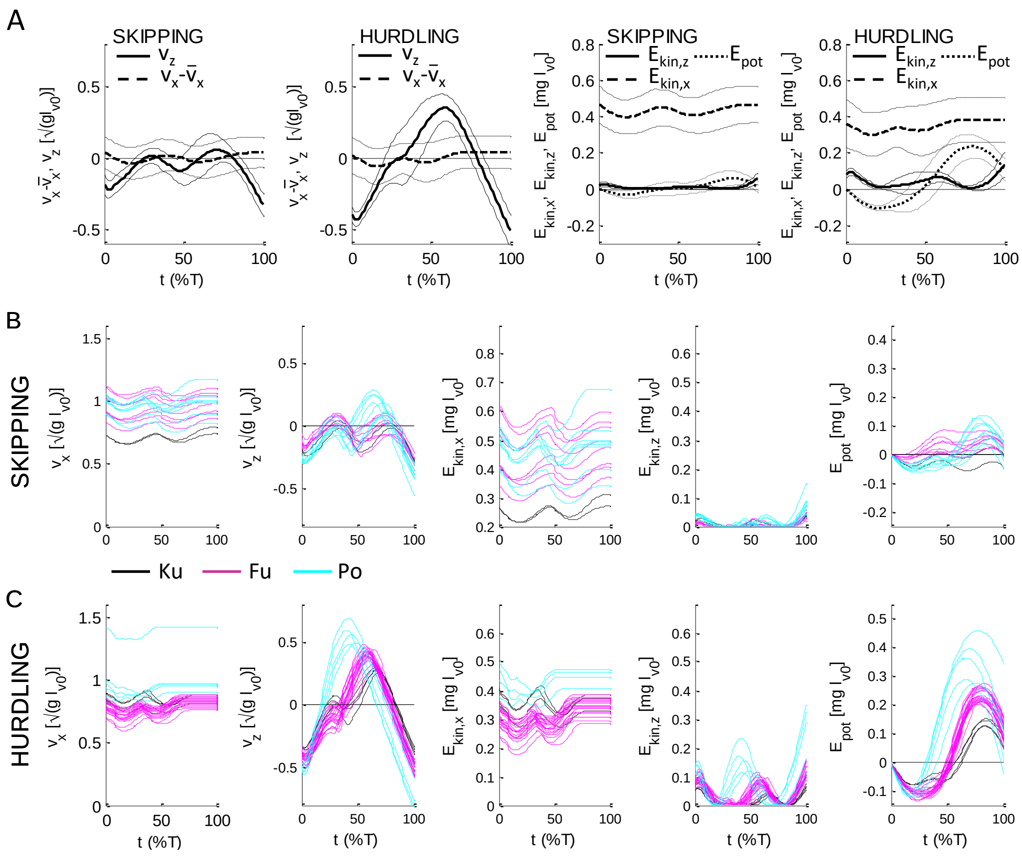

Fig. S2. Velocity and energetics of the CoM during skipping and hurdling. A) Mean ±SD of the time courses of (from left to right) antorad velocity v_x minus its mean during the stride $\bar{v}_x$ (dashed lines) as well as the vertical velocity v_z(solid lines) for skipping and for hurdling. The kinetic energies $E_{kin,x}$ (dashed lines), $E_{kin,z}$ (solid lines), and potential energy E_{pot} (dotted lines) for skipping and hurdling. B,C) For skipping (B) and hurdling (C) for $v_{x,z}$ and for $E_{kin,x,z}$ and E_{pot} the tracings for each trial. Macaques: Ku - black; Fu – magenta; Po - cyan.

Journal of Experimental Biology • Supplementary information

Table S1. Comparison of global parameters between the leading and trailing leg for skipping and hurdling: timing, kinetics, leg properties, energetics. (Probabilities of GLM with repetitions.)

	SKIPPING					HURDLING				
Variables	tr-le	tr-le*v_{Fr}	tr-le*ani	v_{Fr}	ani	tr-le	tr-le*v_{Fr}	tr-le*ani	v_{Fr}	ani
t_c $\left[\sqrt{g/l_{e0}}\right]$	n.s.	n.s.	n.s.	9.5E-10	n.s.	1.8E-15	1.5E-08	4.3E-02	2.4E-08	n.s.
t_{da} $\left[\sqrt{g/l_{e0}}\right]$	1.0E-08	9.9E-04	n.s.	7.1E-04	9.9E-04	1.0E-27	1.5E-05	1.6E-09	7.1E-02	n.s.
$\beta_{eleg-TD}$ (deg)	5.2E-05	3.5E-02	n.s.	n.s.	n.s.	1.1E-16	n.s.	n.s.	n.s.	n.s.
$\beta_{eleg-LO}$ (deg)	8.3E-05	n.s.	n.s.	n.s.	n.s.	7.0E-24	1.1E-03	3.2E-03	9.0E-08	9.4E-08
$\beta_{vleg-TD}$ (deg)	5.3E-05	3.1E-02	n.s.	n.s.	n.s.	6.9E-17	n.s.	n.s.	n.s.	n.s.
$\beta_{vleg-LO}$ (deg)	5.6E-05	n.s.	n.s.	n.s.	n.s.	7.8E-24	2.5E-03	1.8E-03	4.4E-08	2.7E-07
$(l_e - l_{e0})_{TD}$ $[l_{e0}]$	n.s.	n.s.	n.s.	n.s.	n.s.	2.2E-05	n.s.	n.s.	n.s.	n.s.
$(l_e - l_{e0})_{min}$ $[l_{e0}]$	5.9E-03	n.s.	n.s.	n.s.	n.s.	7.2E-23	n.s.	1.8E-07	n.s.	n.s.
$(l_e - l_{e0})_{LO}$ $[l_{e0}]$	n.s.	n.s.	n.s.	5.6E-04	9.7E-03	3.2E-08	n.s.	n.s.	3.1E-08	2.3E-03
$(l_v - l_{v0})_{TD}$ $[l_{v0}]$	n.s.	n.s.	n.s.	2.8E-02	6.8E-03	4.3E-06	1.4E-04	n.s.	4.3E-02	3.7E-03
$(l_v - l_{v0})_{min}$ $[l_{v0}]$	9.0E-03	n.s.	n.s.	n.s.	2.7E-02	1.3E-06	2.8E-03	n.s.	6.2E-05	5.6E-04
$(l_v - l_{v0})_{LO}$ $[l_{v0}]$	n.s.	n.s.	n.s.	1.4E-02	n.s.	2.6E-09	n.s.	n.s.	1.4E-04	2.2E-02
x_{CoP1} $[l_{e0}]$	n.s.	n.s.	n.s.	n.s.	n.s.	n.s.	n.s.	n.s.	n.s.	n.s.
x_{CoP2} $[l_{e0}]$	n.s.	n.s.	n.s.	n.s.	n.s.	8.6E-13	n.s.	n.s.	7.1E-04	5.6E-05
$\beta_{tru-TD}(deg)$	n.s.	n.s.	n.s.	n.s.	5.5E-03	3.6E-10	1.3E-05	1.2E-06	1.2E-09	2.6E-08
$\beta_{tru-min}(deg)$	n.s.	n.s.	n.s.	n.s.	n.s.	n.s.	n.s.	n.s.	3.2E-08	1.3E-05
$\beta_{tru-max}(deg)$	n.s.	n.s.	n.s.	n.s.	n.s.	n.s.	2.4E-07	n.s.	5.6E-12	1.3E-09
$\beta_{tru-LO}(deg)$	2.4E-03	n.s.	n.s.	n.s.	n.s.	2.0E-08	n.s.	n.s.	2.2E-11	3.0E-07
F_{pxa} $[mg]$	n.s.	n.s.	n.s.	n.s.	n.s.	n.s.	n.s.	n.s.	n.s.	n.s.
F_{pym} $[mg]$	n.s.	n.s.	n.s.	n.s.	5.7E-07	n.s.	n.s.	n.s.	2.1E-06	6.8E-06
F_{pz} $[mg]$	n.s.	n.s.	n.s.	2.6E-02	n.s.	n.s.	2.8E-04	n.s.	1.2E-09	4.4E-02
$Skew$ [ ]	2.8E-03	n.s.	2.1E-03	n.s.	2.1E-03	1.3E-16	n.s.	6.1E-03	n.s.	n.s.
$Kurtosis$ [ ]	1.3E-02	n.s.	n.s.	n.s.	n.s.	8.2E-16	n.s.	n.s.	n.s.	n.s.
$p_x[m\sqrt{gl_{v0}}]$	n.s.	n.s.	n.s.	n.s.	n.s.	n.s.	n.s.	n.s.	n.s.	n.s.
$p_{xa}[m\sqrt{gl_{v0}}]$	n.s.	n.s.	n.s.	n.s.	n.s.	n.s.	n.s.	n.s.	n.s.	n.s.
$p_{xp}[m\sqrt{gl_{v0}}]$	n.s.	n.s.	n.s.	n.s.	n.s.	n.s.	n.s.	n.s.	n.s.	n.s.
$p_y[m\sqrt{gl_{v0}}]$	n.s.	n.s.	n.s.	1.4E-03	3.3E-06	n.s.	n.s.	n.s.	5.1E-03	2.1E-05
$p_{ym}[m\sqrt{gl_{v0}}]$	n.s.	n.s.	n.s.	n.s.	n.s.	n.s.	n.s.	n.s.	n.s.	5.1E-06
$p_{yl}[m\sqrt{gl_{v0}}]$	n.s.	n.s.	n.s.	1.1E-02	4.2E-04	n.s.	n.s.	n.s.	3.8E-03	7.8E-03
$p_z[m\sqrt{gl_{v0}}]$	n.s.	n.s.	n.s.	7.1E-04	6.0E-06	n.s.	n.s.	n.s.	n.s.	n.s.
$p_z - mg\ t_c$	n.s.	n.s.	n.s.	2.3E-05	1.4E-04	8.3E-03	3.0E-03	n.s.	1.4E-04	n.s.
k_e $[mg/l_{e0}]$	n.s.	n.s.	n.s.	n.s.	n.s.	4.4E-10	1.8E-02	1.1E-02	n.s.	3.3E-02
D_e $[mg/\sqrt{gl_{e0}}]$	8.2E-03	n.s.	n.s.	n.s.	n.s.	3.1E-18	n.s.	1.1E-03	n.s.	n.s.
k_v $[mg/l_{v0}]$	n.s.	n.s.	n.s.	n.s.	n.s.	n.s.	n.s.	1.2E-02	n.s.	4.2E-07
D_v $[mg/\sqrt{gl_{v0}}]$	3.4E-03	n.s.	n.s.	n.s.	n.s.	4.2E-18	n.s.	n.s.	n.s.	n.s.

x_{vpp} $[l_{e0}]$	2.4E-06	n.s.	n.s.	n.s.	n.s.	8.0E-15	n.s.	n.s.	1.8E-02	n.s.
z_{vpp} $[l_{e0}]$	n.s.	n.s.	n.s.	5.6E-03	n.s.	9.6E-24	n.s.	2.1E-03	n.s.	n.s.
$xw_{vpp}[l_{e0}]$	2.8E-02	n.s.	n.s.	n.s.	n.s.	1.9E-11	n.s.	n.s.	n.s.	n.s.
L_{vy} $[m\, l_{v0} \sqrt{g l_{v0}}]$	n.s.	n.s.	n.s.	n.s.	n.s.	4.1E-07	n.s.	n.s.	1.9E-05	n.s.
W_{eax} $[mg\, l_{e0}]$	n.s.	n.s.	n.s.	4.2E-03	1.0E-02	1.8E-19	n.s.	1.3E-04	n.s.	n.s.
W_{etan} $[mg\, l_{e0}]$	2.6E-06	1.0E-04	1.3E-04	n.s.	n.s.	1.2E-14	7.1E-04	n.s.	n.s.	8.0E-06
W_{vax} $[mg\, l_{v0}]$	n.s.	n.s.	n.s.	4.2E-04	n.s.	2.0E-21	7.6E-03	1.6E-03	n.s.	8.5E-04
W_{vtan} $[mg\, l_{v0}]$	7.5E-08	2.8E-04	n.s.	n.s.	n.s.	3.6E-21	n.s.	1.8E-03	n.s.	2.9E-06
$\Delta E_{kin,x}$ $[mg\, l_{v0}]$	n.s.	n.s.	n.s.	n.s.	n.s.	3.7E-10	n.s.	n.s.	n.s.	n.s.
$\Delta E_{kin,z}$ $[mg\, l_{v0}]$	n.s.	n.s.	1.2E+02	5.6E-04	1.3E-02	3.8E-23	n.s.	3.1E-02	9.1E-06	n.s.
ΔE_{pot} $[mg\, l_{v0}]$	1.5E-02	3.4E-03	1.5E-02	n.s.	n.s.	1.3E-31	n.s.	4.9E-06	1.2E-05	3.6E-07
ΔE_{ext} $[mg\, l_{v0}]$	4.7E-05	6.2E-03	2.5E-03	n.s.	n.s.	2.6E-27	n.s.	1.8E-05	7.8E-03	1.7E-02

Probabilities listed for the comparison with repetition between the trailing and leading leg (tr-le), the interaction with Froude speed (tr-le $*v_{Fr}$) and the interaction with individual (tr-le*ani), as well as the main factors Froude speed (v_{Fr}) and individual (ani). Variables: t_c, contact time; t_{da}, aerial time (>0) or double support (<=0); $\beta_{eleg|vleg}$, leg angle of the effective|virtual leg; $l_{e|v}$, length of effective|virtual leg; $x_{CoP1|CoP2}$, posterior/anterior foot contact length; β_{tru}, inclination of trunk; $F_{pxa|pym|pz}$, peak anteriad|mediad|vertical ground reaction force; $p_{x|xa|xp}$, impulse posteri-anterioriad|anteriad|posteriad; $p_{y|ym|yl}$, impulse lateri-mediad|mediad|laterad; p_z, vertical impulse; $p_z - mg\, t_c$, vertical impulse minus impulse due to gravitation; $k_{e|v}$, stiffness of effective|virtual leg; $D_{e|v}$, damping (>0) of effective|virtual leg; $x|z_{vpp}$, locus of virtual pivot point with respect to CoM; xw_{vpp}, with of virtual pivot point; L_{vy}, rotational impulse of virtual leg; $W_{e|vax|tan}$, work of effective and virtual leg in axial and tangential direction; $\Delta E_{kin,x|z}$, change of kinetic energy of CoM; ΔE_{pot}, change of potential energy of CoM; ΔE_{ext}, change of external energy of CoM. Abbreviations in units: l_{e0}, length of effective leg; l_{v0}, length of virtual leg; m, body mass; g, gravitational acceleration; TD, touch down, LO, lift off; min, minimum; max, maximum. For all comparisons Bonferroni f = 141; n.s., p>0.05.

Table S2. Comparison of global parameters between the skipping and hurdling for the trailing and leading leg: timing, kinetics, leg properties, energetics. (Probabilities of univariate GLM)

	TRAILING			LEADING		
	sk-hu	sk-hu *v_{Fr}	sk-hu*ani	sk-hu	sk-hu *v_{Fr}	sk-hu*ani
$T\ [\sqrt{g/l_{e0}}]$				7.3E-08	5.3E-09	1.0E-04
$t_c\ [\sqrt{g/l_{e0}}]$	1.9E-02	6.8E-15	n.s.	n.s	3.5E-19	1.4E-04
$t_{da}\ [\sqrt{g/l_{e0}}]$	1.5E-14	n.s.	4.1E-05	1.3E-25	2.2E-13	1.3E-14
$\beta_{eleg-TD}\ (deg)$	n.s.	n.s.	n.s.	9.6E-16	n.s..	n.s.
$\beta_{eleg-LO}\ (deg)$	9.2E-05	1.5E-07	7.2E-05	1.2E-24	1.7E-02	7.4E-06
$\beta_{vleg-TD}\ (deg)$	n.s.	n.s.	n.s.	7.2E-08	n.s.	n.s.
$\beta_{vleg-LO}\ (deg)$	n.s.	3.6E-11	6.1E-06	1.4E-16	n.s.	4.4E-06
$(l_e - l_{e0})_{TD}\ [l_{e0}]$	n.s.	n.s.	n.s.	n.s.	n.s.	n.s.
$(l_e - l_{e0})_{min}\ [l_{e0}]$	6.6E-18	n.s.	7.1E-04	n.s.	1.9E-06	1.8E-06
$(l_e - l_{e0})_{LO}\ [l_{e0}]$	n.s.	8.8E-06	n.s.	2.7E-15	1.7E-13	1.0E-14
$(l_v - l_{v0})_{TD}\ [l_{v0}]$	n.s.	n.s.	n.s.	4.0E-11	2.4E-08	9.3E-08
$(l_v - l_{v0})_{min}\ [l_{v0}]$	2.5E-12	n.s.	5.4E-03	3.4E-13	5.2E-10	1.3E-05
$(l_v - l_{v0})_{LO}\ [l_{v0}]$	n.s.	3.0E-03	n.s.	9.8E-17	4.5E-08	1.8E-07
$x_{CoP1}\ [l_{e0}]$	n.s.	n.s.	n.s.	n.s.	n.s.	n.s.
$x_{CoP2}\ [l_{e0}]$	n.s.	2.7E-02	3.0E-02	n.s.	1.1E-03	6.0E-08
$\beta_{tru-TD}(deg)$	5.3E-05	8.8E-10	6.3E-13	1.3E-08	2.9E-10	1.0E-09
$\beta_{tru-min}(deg)$	5.9E-07	1.1E-09	3.7E-09	1.0E-07	9.2E-09	2.7E-06
$\beta_{tru-max}(deg)$	2.0E-07	5.4E-10	5.3E-12	1.4E-09	2.8E-13	4.5E-09
$\beta_{tru-LO}(deg)$	4.8E-08	2.1E-10	1.9E-07	4.3E-10	1.7E-13	2.2E-08
$F_{pxa}\ [mg]$	5.3E-05	8.8E-10	6.3E-13	1.3E-08	2.9E-10	1.0E-09
$F_{pym}\ [mg]$	5.9E-07	1.1E-09	3.7E-09	1.0E-07	9.2E-09	2.7E-06
$F_{pz}\ [mg]$	2.0E-07	5.4E-10	5.3E-12	1.4E-09	2.8E-13	4.5E-09
$Skew$ [ ]	4.8E-08	2.1E-10	1.9E-07	4.3E-10	1.7E-13	2.2E-08
$Kurtosis$ [ ]	7.1E-09	n.s.	n.s.	n.s.	n.s.	n.s.
$p_x[m\sqrt{gl_{v0}}]$	n.s.	n.s.	n.s.	n.s.	n.s.	n.s.
$p_{xa}[m\sqrt{gl_{v0}}]$	n.s.	n.s.	n.s.	n.s.	n.s.	n.s.
$p_{xp}[m\sqrt{gl_{v0}}]$	n.s.	n.s.	n.s.	n.s.	n.s.	n.s.
$p_y[m\sqrt{gl_{v0}}]$	n.s.	2.1E-02	3.3E-05	n.s.	2.0E-03	3.0E-08
$p_{ym}[m\sqrt{gl_{v0}}]$	n.s.	5.4E-03	2.9E-06	n.s.	2.1E-03	3.4E-09
$p_{yl}[m\sqrt{gl_{v0}}]$	n.s.	n.s.	2.1E-02	n.s.	2.0E-02	9.9E-04
$p_z[m\sqrt{gl_{v0}}]$	7.1E-04	n.s.	n.s.	2.3E-09	5.6E-04	5.6E-04
$p_z - mg\ t_c$	1.7E-02	2.2E-06	n.s.	2.3E-11	9.5E-06	7.1E-04
$k_e\ [mg/l_{e0}]$	1.3E-07	3.0E-02	7.4E-06	6.8E-03	n.s.	n.s.
$D_e\ [mg/\sqrt{gl_{e0}}]$	n.s.	4.5E-03	n.s.	4.0E-06	n.s.	2.5E-03
$k_v\ [mg/l_{v0}]$	1.9E-06	n.s.	5.2E-06	3.4E-14	n.s.	n.s.
$D_v\ [mg/\sqrt{gl_{v0}}]$	3.1E-08	n.s.	n.s.	5.6E-04	n.s.	n.s.
$x_{vpp}\ [l_{e0}]$	n.s.	n.s.	n.s.	3.2E-06	n.s.	2.8E-04
$z_{vpp}\ [l_{e0}]$	4.2E-04	n.s.	n.s.	1.5E-12	7.0E-05	n.s.
$xw_{vpp}[l_{e0}]$	4.6E-05	n.s.	n.s.	7.3E-05	2.3E-03	7.8E-06

Journal of Experimental Biology • Supplementary information

L_{vy} $[m\, l_{v0}\sqrt{g l_{v0}}]$	n.s.	1.3E-03	n.s.	n.s.	4.2E-04	9.0E-03
W_{eax} $[mg\ l_{e0}]$	9.5E-07	2.2E-02	5.5E-03	7.5E-15	1.8E-03	2.0E-06
W_{etan} $[mg\ l_{e0}]$	n.s.	2.8E-04	2.3E-05	6.4E-19	2.4E-03	4.0E-07
W_{vax} $[mg\ l_{v0}]$	3.1E-05	9.3E-03	n.s.	7.4E-24	1.4E-04	1.3E-08
W_{vtan} $[mg\ l_{v0}]$	n.s.	2.0E-08	4.7E-05	2.3E-11	n.s.	3.3E-07
$\Delta E_{kin,x}$ $[mg\ l_{v0}]$	n.s.	n.s.	n.s.	n.s.	n.s.	n.s.
$\Delta E_{kin,z}$ $[mg\ l_{v0}]$	8.1E-07	1.4E-06	n.s.	4.9E-06	1.2E-07	4.2E-03
ΔE_{pot} $[mg\ l_{v0}]$	4.7E-08	2.0E-08	6.9E-05	2.3E-28	2.6E-07	2.9E-10
ΔE_{ext} $[mg\ l_{v0}]$	9.6E-06	4.2E-04	n.s.	3.7E-22	4.7E-03	2.3E-08
$Congruity$ [ ]				1.3E-13	3.3E-02	n.s.
$Recovery$ [%]				1.9E-02	n.s.	n.s.
CoT $[mg]$				3.7E-16	n.s.	2.1E-02

Probabilities listed for the univariate comparison between the skipping and hurdling (sk-hu) for the trailing and leading leg, the interaction with Froude speed (sk-hu $*v_{Fr}$) and the interaction with individual (sk-hu *ani). Variables: T, stride period; t_c, contact time; t_{da}, aerial time (>0) or double support (<=0); $\beta_{eleg|vleg}$, leg angle of the effective|virtual leg; $l_{e|v}$, length of effective|virtual leg; $x_{CoP1|\,CoP2}$, posterior/ anterior foot contact length; β_{tru}, inclination of trunk; $F_{pxa|pym|pz}$, peak anteriad|mediad|vertical ground reaction force; $p_{x|xa|xp}$, impulse posteri-anterioriad|anteriad|posteriad; $p_{y|ym|yl}$, impulse lateri-mediad|mediad|laterad; p_z, vertical impulse; $p_z - mg\ t_c$, vertical impulse minus impulse due to gravitation; $k_{e|v}$, stiffness of effective|virtual leg; $D_{e|v}$, damping (>0) of effective|virtual leg; $x|z_{vpp}$, locus of virtual pivot point with respect to CoM; xw_{vpp}, with of virtual pivot point; L_{vy}, rotational impulse of virtual leg; $W_{e|vax|tan}$, work of effective and virtual leg in axial and tangential direction; $\Delta E_{kin,x|z}$, change of kinetic energy of CoM; ΔE_{pot}, change of potential energy of CoM; ΔE_{ext}, change of external energy of CoM; CoT, mechanical cost of transport of CoM; TD, touch down, LO, lift off; min, minimum; max, maximum. Abbreviations in units: l_{e0}, length of effective leg; l_{v0}, length of virtual leg; m, body mass; g, gravitational acceleration. The probabilities referring to strides are listed at the leading leg. For all comparisons Bonferroni f = 141; n.s., p>0.05.

Table S3. Comparison of global parameters between the leading and trailing leg and skipping and hurdling: timing, kinetics, leg properties, energetics. (Probabilities of t- or Wilcoxon test)

		SKIPPING					HURDLING					p_{trlesk}	p_{trlehu}*
Variable		Mean	Std	Med	Min	Max	Mean	Std	Med	Min	Max	p_{skhutr}	p_{skhule}
$T\ \left[\sqrt{g/l_{e0}}\right]$	stride	2.416	±0.212	2.414	2.026	2.799	2.652	±0.185	2.653	2.197	3.094		1.66E-03
$t_c\ \left[\sqrt{g/l_{e0}}\right]$	trail	1.144	±0.146	1.095	0.970	1.441	1.218	±0.138	1.218	0.887	1.541	n.s.	3.80E-05
	lead	1.102	±0.164	1.069	0.887	1.466	1.068	±0.067	1.069	0.970	1.268	n.s.	n.s.
$t_{da}\ \left[\sqrt{g/l_{e0}}\right]$	trail	-0.042	±0.043	-0.039	-0.139	0.000	-0.350	±0.142	-0.398	-0.527	0.000	8.15E-05	1.30E-18
	lead	0.298	±0.251	0.174	0.025	0.721	0.914	±0.235	0.870	0.531	1.580	3.43E-11	9.08E-11
$\beta_{eleg-TD}$ (deg)	trail	-27.07	±3.96	-28.45	-31.73	-18.86	-25.90	±4.13	-25.45	-36.73	-19.46	n.s.	3.52E-06
	lead	-28.82	±2.11	-29.08	-31.87	-25.56	-37.26	±2.28	-36.98	-42.16	-32.09	n.s.	2.30E-08
$\beta_{eleg-LO}$ (deg)	trail	35.51	±4.80	35.95	25.65	43.69	31.50	±4.03	32.55	20.19	36.74	1.89E-03	3.52E-06
	lead	27.43	±3.46	26.99	21.14	34.40	11.69	±3.42	10.95	5.62	20.01	1.14E-02	2.30E-08
$\beta_{vleg-TD}$ (deg)	trail	-17.08	±3.23	-17.62	-22.22	-12.17	-14.44	±3.22	-13.48	-20.66	-9.85	1.89E-03	3.52E-06
	lead	-21.05	±1.86	-21.12	-23.64	-18.12	-25.84	±2.60	-25.34	-32.34	-22.11	3.32E-02	4.38E-07
$\beta_{vleg-LO}$ (deg)	trail	31.40	±3.85	31.88	24.08	37.71	30.83	±4.75	32.50	17.53	36.82	1.61E-03	1.08E-20
	lead	23.59	±4.11	24.11	17.02	31.21	12.77	±3.61	13.24	5.92	18.68	n.s.	7.71E-08
$(l_e - l_{e0})_{TD}\ [l_{e0}]$	trail	0.972	±0.031	0.968	0.912	1.047	0.949	±0.025	0.944	0.911	1.005	n.s.	8.23E-05
	lead	0.984	±0.028	0.981	0.939	1.039	0.979	±0.034	0.972	0.929	1.055	1.80E-02	n.s.
$(l_e - l_{e0})_{min}\ [l_{e0}]$	trail	0.859	±0.019	0.859	0.829	0.892	0.763	±0.029	0.760	0.715	0.833	1.13E-02	3.61E-17
	lead	0.890	±0.030	0.889	0.838	0.930	0.883	±0.024	0.882	0.829	0.942	2.94E-08	n.s.
$(l_e - l_{e0})_{LO}\ [l_{e0}]$	trail	1.088	±0.083	1.071	0.980	1.286	1.063	±0.057	1.066	0.924	1.169	n.s.	3.52E-06
	lead	1.073	±0.077	1.040	0.969	1.172	1.148	±0.040	1.138	1.087	1.264	n.s.	1.31E-04
$(l_v - l_{v0})_{TD}\ [l_{v0}]$	trail	0.964	±0.027	0.961	0.917	1.007	0.952	±0.029	0.951	0.901	1.045	n.s.	5.46E-04
	lead	0.977	±0.028	0.974	0.918	1.033	0.917	±0.047	0.899	0.852	1.011	n.s.	3.03E-04
$(l_v - l_{v0})_{min}\ [l_{v0}]$	trail	0.900	±0.015	0.903	0.873	0.933	0.836	±0.029	0.834	0.788	0.897	2.21E-03	2.37E-05
	lead	0.926	±0.019	0.931	0.887	0.949	0.869	±0.039	0.860	0.799	0.939	1.39E-07	3.46E-05
$(l_v - l_{v0})_{LO}\ [l_{v0}]$	trail	1.073	±0.048	1.058	1.010	1.193	1.068	±0.042	1.082	0.976	1.133	n.s.	3.52E-06
	lead	1.070	±0.038	1.072	1.021	1.123	1.134	±0.021	1.132	1.108	1.201	n.s.	3.12E-07
$x_{CoP1}\ [l_{e0}]$	trail	0.043	±0.027	0.041	0.005	0.097	0.039	±0.029	0.034	0.000	0.095	4.16E-02	n.s.
	lead	0.024	±0.020	0.017	0.003	0.073	0.039	±0.032	0.035	0.001	0.154	n.s.	n.s.
$x_{CoP2}\ [l_{e0}]$	trail	0.148	±0.052	0.147	0.049	0.236	0.118	±0.056	0.103	0.040	0.275	n.s.	3.52E-06
	lead	0.179	±0.032	0.180	0.124	0.222	0.201	±0.046	0.183	0.147	0.317	n.s.	n.s.
$\beta_{tru-TD}(deg)$	trail	29.04	±5.90	27.10	39.76	19.23	32.91	±5.75	35.04	40.20	20.83	n.s.	3.88E-05
	lead	28.20	±4.82	28.72	35.62	19.64	35.59	±7.99	39.03	46.08	19.74	n.s.	4.06E-03
$\beta_{tru-min}(deg)$	trail	30.07	±5.99	28.86	40.21	20.67	36.72	±7.58	39.55	50.25	21.84	n.s.	n.s.
	lead	28.85	±4.69	30.26	36.28	21.45	36.63	±7.71	39.54	50.52	21.11	5.03E-03	2.43E-03
$\beta_{tru-max}(deg)$	trail	26.79	±5.09	25.50	35.43	19.10	32.18	±6.62	34.85	39.29	19.33	1.71E-02	n.s.
	lead	25.22	±4.76	27.09	32.98	17.73	32.53	±7.91	36.18	43.13	17.80	7.14E-03	3.27E-03
$\beta_{tru-LO}(deg)$	trail	27.69	±5.19	27.02	36.15	20.05	35.75	±8.51	38.49	50.25	19.33	1.89E-03	6.70E-09
	lead	25.30	±4.76	27.22	33.31	18.32	32.75	±7.60	36.18	43.13	18.55	2.82E-03	2.43E-03
$F_{pxa}\ [mg]$	trail	0.213	±0.040	0.209	0.148	0.298	0.250	±0.029	0.253	0.178	0.304	n.s.	3.64E-02
	lead	0.218	±0.053	0.213	0.141	0.380	0.271	±0.031	0.275	0.206	0.329	6.22E-03	8.02E-05
$F_{pym}\ [mg]$	trail	-0.170	±0.055	-0.164	-0.260	-0.072	-0.155	±0.047	-0.151	-0.298	-0.089	n.s.	n.s.
	lead	-0.138	±0.093	-0.113	-0.300	-0.006	-0.163	±0.058	-0.142	-0.320	-0.069	n.s.	n.s.

Journal of Experimental Biology • Supplementary information

F_{pz} $[mg]$	trail	1.618	±0.148	1.581	1.401	1.905	1.927	±0.270	1.817	1.594	2.606	n.s.	n.s.
	lead	1.571	±0.224	1.648	1.155	1.875	1.892	±0.163	1.939	1.610	2.211	6.67E-05	6.67E-05
$Skew$ [ ]	trail	0.312	±0.063	0.306	0.190	0.417	0.398	±0.058	0.405	0.233	0.510	3.68E-02	3.52E-06
	lead	0.268	±0.066	0.243	0.158	0.391	0.179	±0.056	0.187	0.055	0.258	1.15E-04	3.03E-04
$Kurtosis$ [ ]	trail	-0.703	±0.068	-0.695	-0.860	-0.593	-0.490	±0.113	-0.457	-0.717	-0.325	1.71E-02	2.74E-15
	lead	-0.784	±0.083	-0.789	-0.911	-0.628	-0.834	±0.049	-0.834	-0.942	-0.736	1.33E-06	n.s.
$p_x[m\sqrt{gl_{v0}}]$	trail	-0.005	±0.032	-0.001	-0.084	0.057	0.013	±0.030	0.010	-0.045	0.085	n.s.	n.s.
	lead	0.006	±0.040	-0.006	-0.048	0.119	0.017	±0.028	0.020	-0.056	0.083	n.s.	n.s.
$p_{xa}[m\sqrt{gl_{v0}}]$	trail	0.083	±0.017	0.079	0.055	0.114	0.102	±0.024	0.096	0.061	0.162	n.s.	n.s.
	lead	0.086	±0.025	0.083	0.053	0.156	0.102	±0.017	0.098	0.083	0.165	2.31E-02	8.76E-03
$p_{xp}[m\sqrt{gl_{v0}}]$	trail	-0.088	±0.020	-0.084	-0.140	-0.057	-0.089	±0.018	-0.089	-0.141	-0.051	n.s.	n.s.
	lead	-0.080	±0.018	-0.087	-0.102	-0.037	-0.085	±0.019	-0.084	-0.138	-0.048	n.s.	n.s.
$p_y[m\sqrt{gl_{v0}}]$	trail	0.045	±0.044	0.028	-0.014	0.107	0.040	±0.021	0.042	-0.008	0.075	n.s.	n.s.
	lead	0.034	±0.061	0.032	-0.049	0.158	0.044	±0.039	0.034	-0.005	0.132	n.s.	n.s.
$p_{ym}[m\sqrt{gl_{v0}}]$	trail	0.063	±0.035	0.050	0.021	0.116	0.061	±0.016	0.065	0.027	0.096	n.s.	n.s.
	lead	0.054	±0.047	0.046	0.000	0.161	0.060	±0.032	0.046	0.024	0.134	n.s.	n.s.
$p_{yl}[m\sqrt{gl_{v0}}]$	trail	-0.017	±0.009	-0.017	-0.035	-0.008	-0.022	±0.008	-0.022	-0.042	-0.003	n.s.	7.16E-03
	lead	-0.020	±0.015	-0.015	-0.049	-0.002	-0.015	±0.009	-0.015	-0.035	-0.001	n.s.	n.s.
$p_z[m\sqrt{gl_{v0}}]$	trail	1.156	±0.129	1.119	0.897	1.412	1.342	±0.143	1.285	1.101	1.613	n.s.	n.s.
	lead	1.120	±0.141	1.152	0.938	1.297	1.334	±0.115	1.338	1.110	1.638	3.90E-04	2.59E-05
$p_z - mg\ t_c$	trail	0.017	±0.161	0.025	-0.214	0.296	0.234	±0.304	0.144	-0.195	0.897	n.s.	1.62E-02
$[m\sqrt{gl_{v0}}]$	lead	0.023	±0.353	0.015	-0.487	0.509	0.535	±0.213	0.571	0.158	0.884	3.32E-02	3.46E-05
k_e $[mg/l_{e0}]$	trail	9.688	±2.166	9.209	6.347	14.071	7.156	±1.511	6.657	5.398	11.526	1.71E-02	1.97E-05
	lead	11.822	±1.260	11.708	9.009	14.673	10.064	±1.528	10.311	6.596	12.019	1.05E-04	1.22E-03
D_e $[mg/\sqrt{gl_{e0}}]$	trail	0.000	±0.441	-0.008	-1.034	0.732	0.246	±0.212	0.253	-0.350	0.732	9.81E-04	3.52E-06
	lead	-0.524	±0.658	-0.324	-1.666	0.357	-1.290	±0.447	-1.389	-2.040	-0.243	n.s.	8.19E-04
k_v $[mg/l_{v0}]$	trail	14.123	±3.149	13.788	8.589	18.727	10.338	±2.842	9.857	7.399	17.942	n.s.	n.s.
	lead	16.528	±2.293	16.037	11.484	21.100	9.811	±1.565	9.995	7.335	13.073	3.03E-04	3.75E-08
D_v $[mg/\sqrt{gl_{v0}}]$	trail	-0.682	±0.397	-0.764	-1.217	0.039	-0.034	±0.277	-0.047	-0.814	0.554	2.21E-03	3.52E-06
	lead	-1.463	±0.685	-1.579	-2.803	-0.266	-2.313	±0.568	-2.506	-3.117	-0.966	1.07E-05	3.03E-04
x_{vpp} $[l_{e0}]$	trail	-0.108	±0.048	-0.123	-0.193	-0.004	-0.162	±0.069	-0.173	-0.286	-0.042	9.81E-04	3.52E-06
	lead	0.035	±0.052	0.042	-0.069	0.132	0.111	±0.057	0.096	0.025	0.215	n.s.	5.91E-04
z_{vpp} $[l_{e0}]$	trail	0.270	±0.146	0.257	0.032	0.542	0.432	±0.094	0.432	0.260	0.642	9.86E-03	3.52E-06
	lead	0.090	±0.177	0.040	-0.104	0.612	-0.205	±0.076	-0.211	-0.336	-0.011	7.55E-04	9.78E-08
$xw_{vpp}[l_{e0}]$	trail	0.190	±0.043	0.196	0.111	0.266	0.329	±0.105	0.336	0.141	0.516	2.59E-03	3.52E-06
	lead	0.138	±0.054	0.146	0.063	0.219	0.099	±0.024	0.094	0.064	0.152	5.54E-05	n.s.
L_{vy} $[m\ l_{v0}\sqrt{gl_{v0}}]$	trail	-0.171	±0.287	-0.022	-0.822	0.108	-0.114	±0.139	-0.157	-0.338	0.181	n.s.	2.60E-05
	lead	0.092	±0.271	0.037	-0.201	1.047	0.171	±0.206	0.108	-0.106	0.753	n.s.	n.s.
W_{ax} $[mg\ l_{e0}]$	trail	0.019	±0.071	0.001	-0.085	0.157	-0.081	±0.064	-0.076	-0.214	0.067	7.42E-03	3.52E-06
	lead	0.063	±0.078	0.035	-0.037	0.190	0.211	±0.053	0.206	0.095	0.306	5.54E-05	1.19E-06
W_{tan} $[mg\ l_{e0}]$	trail	0.005	±0.080	0.010	-0.131	0.145	0.054	±0.070	0.028	-0.043	0.260	2.87E-02	2.68E-12
	lead	0.082	±0.029	0.075	0.048	0.163	0.246	±0.066	0.268	0.085	0.373	n.s.	8.62E-13
W_{vax} $[mg\ l_{v0}]$	trail	0.065	±0.042	0.063	-0.003	0.133	0.003	±0.047	0.012	-0.152	0.105	n.s.	3.52E-06
	lead	0.086	±0.041	0.081	0.017	0.156	0.293	±0.064	0.306	0.135	0.394	1.38E-04	3.32E-08

Journal of Experimental Biology • Supplementary information

W_{vtan} $[mg\ l_{v0}]$	trail	-0.069	±0.045	-0.079	-0.132	0.006	-0.080	±0.033	-0.092	-0.118	-0.008	7.00E-04	3.52E-06
	lead	0.023	±0.034	0.023	-0.044	0.086	0.088	±0.034	0.083	0.019	0.140	n.s.	1.44E-05
$\Delta E_{kin,x}$ $[mg\ l_{v0}]$	trail	-0.024	±0.027	-0.022	-0.087	0.020	-0.039	±0.031	-0.044	-0.099	0.018	1.4E-03	2.6E-06
	lead	0.007	±0.035	-0.003	-0.044	0.112	0.032	±0.027	0.035	-0.041	0.081	n.s.	1.7E-03
$\Delta E_{kin,z}$ $[mg\ l_{v0}]$	trail	-0.010	±0.017	-0.009	-0.038	0.016	-0.047	±0.030	-0.054	-0.072	0.082	7.4E-03	5.8E-24
	lead	0.006	±0.018	0.004	-0.025	0.038	0.039	±0.034	0.034	0.002	0.157	1.6E-06	9.3E-05
ΔE_{pot} $[mg\ l_{v0}]$	trail	-0.001	±0.037	-0.004	-0.053	0.085	-0.046	±0.035	-0.056	-0.099	0.054	4.8E-02	1.3E-28
	lead	0.053	±0.068	0.052	-0.055	0.161	0.294	±0.052	0.293	0.171	0.395	7.8E-05	7.7E-09
ΔE_{ext} $[mg\ l_{v0}]$	trail	-0.035	±0.068	-0.037	-0.136	0.070	-0.132	±0.071	-0.149	-0.224	0.101	5.7E-03	1.2E-06
	lead	0.067	±0.057	0.057	-0.010	0.164	0.365	±0.099	0.359	0.135	0.606	1.8E-05	1.1E-08
$Congruity$ [ ]	stride	0.694	±0.091	0.717	0.522	0.834	0.477	±0.064	0.460	0.367	0.591		1.1E-07
$Recovery$ [%]	stride	3.837	±2.803	2.564	0.816	10.389	6.792	±2.254	7.519	2.093	9.814		1.9E-04
CoT $[mg]$	stride	0.126	±0.015	0.127	0.100	0.155	0.219	±0.034	0.221	0.133	0.313		1.4E-08

T, stride period; t_c, contact time; t_{da}, aerial time (>0) or double support (<=0); $\beta_{eleg|vleg}$, angle of the effective|virtual leg; $l_{e|v}$, length of effective|virtual leg; $x_{CoP1|\,CoP2}$, posterior|anterior foot contact length; β_{tru}, inclination of trunk; $F_{pxa|pym|pz}$, peak anteriad|mediad|vertical ground reaction force; $p_{x|xa|xp}$, impulse posteri-anterioriad|anteriad|posteriad; $p_{y|ym|yl}$, impulse lateri-mediad|mediad|laterad; p_z, vertical impulse; $p_z - mg\,t_c$, vertical impulse minus impulse due to gravitation; $k_{e|v}$, stiffness of effective|virtual leg; $D_{e|v}$, damping (>0) of effective|virtual leg; $x|z_{vpp}$, locus of virtual pivot point with respect to CoM; xw_{vpp}, with of virtual pivot point; L_{vy}, rotational impulse of virtual leg; $W_{e|vax|tan}$, work of effective and virtual leg in axial and tangential direction; $\Delta E_{kin,x|z}$, change of kinetic energy of CoM; ΔE_{pot}, change of potential energy of CoM; ΔE_{ext}, change of external energy of CoM; CoT, mechanical cost of transport of CoM. Abbreviations in units: l_{e0}, length of effective leg; l_{v0}, length of virtual leg; m, body mass; g, gravitational acceleration; TD, touch down, LO, lift off; min, minimum; max, maximum; med, median; $trlesk|trlehu$, comparison trailing leading for skipping/hurdling; $skhutr|\ skhule$, comparison skipping and hurdling for trailing/leading leg; * for quantities referring to complete strides comparison between skipping and hurdling. For all comparisons (t-test, Wilcoxon tests) Bonferroni f = 3; n.s., p>0.05.

Supplementary Materials and Methods

Calculation of velocity and vertical displacement of the CoM from ground reaction forces.

Vertical component

$$v_{vz}(t) = \int_{t=0}^{t} a_z\,dt - \int_{t=0}^{t=t_{LO,lead}} a_z\,dt + v_{vz,TD,lead} \tag{1}$$

$$z_v(t) = z_{vTD,trail} + \int_{t=0}^{t} \left(v_{vz} - \int_{t=0}^{t=t_{LO,lead}} v_{vz}dt\right) dt + \frac{t}{t_{LO,lead}}\left(z_{vLO,lead} - z_{vTD,trail}\right). \tag{2}$$

Horizontal component

$$v_{vx}(t) = \int_{t=0}^{t} a_x\,dt + v_{vx,LO,lead} - \int_{t=0}^{t=t_{LO,lead}} a_x dt. \tag{3}$$
